# Estimating colocalization probability from limited summary statistics

**DOI:** 10.1186/s12859-021-04170-z

**Published:** 2021-05-17

**Authors:** Emily A. King, Fengjiao Dunbar, Justin Wade Davis, Jacob F. Degner

**Affiliations:** AbbVie Genomics Research Center, North Chicago, IL USA

**Keywords:** Colocalization, Genome-wide association study, GWAS, Expression quantitative trait locus, eQTL, GTEx, GWAS catalog

## Abstract

**Background:**

Colocalization is a statistical method used in genetics to determine whether the same variant is causal for multiple phenotypes, for example, complex traits and gene expression. It provides stronger mechanistic evidence than shared significance, which can be produced through separate causal variants in linkage disequilibrium. Current colocalization methods require full summary statistics for both traits, limiting their use with the majority of reported GWAS associations (e.g. GWAS Catalog). We propose a new approximation to the popular coloc method that can be applied when limited summary statistics are available.

Our method (POint EstiMation of Colocalization, POEMColoc) imputes missing summary statistics for one or both traits using LD structure in a reference panel, and performs colocalization using the imputed summary statistics.

**Results:**

We evaluate the performance of POEMColoc using real (UK Biobank phenotypes and GTEx eQTL) and simulated datasets. We show good correlation between posterior probabilities of colocalization computed from imputed and observed datasets and similar accuracy in simulation. We evaluate scenarios that might reduce performance and show that multiple independent causal variants in a region and imputation from a limited subset of typed variants have a larger effect while mismatched ancestry in the reference panel has a modest effect. Further, we find that POEMColoc is a better approximation of coloc when the imputed association statistics are from a well powered study (*e.g.*, relatively larger sample size or effect size). Applying POEMColoc to estimate colocalization of GWAS Catalog entries and GTEx eQTL, we find evidence for colocalization of 150,000 trait-gene-tissue triplets.

**Conclusions:**

We find that colocalization analysis performed with full summary statistics can be closely approximated when only the summary statistics of the top SNP are available for one or both traits. When applied to the full GWAS Catalog and GTEx eQTL, we find that colocalized trait-gene pairs are enriched in tissues relevant to disease etiology and for matches to approved drug mechanisms. POEMColoc R package is available at https://github.com/AbbVie-ComputationalGenomics/POEMColoc.

**Supplementary Information:**

The online version contains supplementary material available at 10.1186/s12859-021-04170-z.

## Background

Genome-wide association studies have identified thousands of disease and trait associated loci but as most GWAS associated loci are non-coding, their functional interpretation remains difficult. eQTL studies are a popular way to follow up on GWAS studies. By providing a link between an associated variant and a gene, they facilitate functional interpretation of GWAS results, especially when associated variants are found in noncoding regions of the genome. A simple approach is to query eQTL datasets for GWAS lead variants to determine if they are significant eQTL for any gene. However, this approach can lead to false positive GWAS-eQTL links as linkage disequilibrium can lead to shared significance at a SNP without the two traits sharing a causal variant. Colocalization analysis reduces false positive results by directly testing competing hypotheses of causal sharing.

Regulatory Trait Concordance (RTC) [1] is an early method for estimating causal sharing that may be applied to situations in which only a GWAS top SNP is known. However, it requires individual-level data for the second dataset (*e.g.* eQTL) and does not actually provide a probability of causal sharing, merely a score that can be used to prioritize the most likely causal links. Coloc [2] and its multi-trait extension moloc [3] are popular colocalization methods with an efficient and easy to use R implementation. They require full summary statistics for both traits and compute the probability of each causal hypothesis using approximate Bayes factors. eCAVIAR [4] is another colocalization method that can also be applied to summary statistics (supplemented with LD information) that has the additional advantage of being able account for more than one causal variant in a region. Enloc [5] is a third approach to colocalization using a Bayesian hierarchical model to compute a regional colocalization probability within an LD block containing a GWAS signal.

One limitation for using most of these colocalization methods is that full summary statistics for GWAS studies are frequently not available. For example, the largest repository of human trait associated variants, the GWAS Catalog, only reports statistics for the top-associated variants in a given study. For this reason, until recently, only simple approaches such as checking the eQTL significance of the reported GWAS variant were possible. Like the POEMColoc method, PICCOLO [6] was recently developed to compute colocalization of signals when only the top SNP is available. PICCOLO first uses PICS [7] to calculate the probability of each SNP being causal, and then calculates the posterior probability of colocalization. However, the reliance on PICS introduces the disadvantage that colocalization analysis will only use SNPs returned by PICS, which is based on 1000 Genomes Phase 1 variants with r^2^ ≥ 0.5 to the top SNP, and the causal probability of these SNPs will be constrained to sum to 1. Therefore, PICCOLO cannot account for the scenario in which one or more of the datasets contains no causal signal. In contrast, POEMColoc does not discard information when full summary statistics are available for one but not both of the traits and does not assume that both traits have a causal variant in the region.

## Results

### Method overview

Here, we propose a method POEMColoc (POint EstiMation of Colocalization) where the explicit goal is to approximate the coloc method [2] under the commonly encountered constraint wherein full summary statistics are unavailable for one or both traits. Consider the situation in which Z-scores ${Z}_{\mathrm{1,1}},\dots ,{Z}_{1,p}$ are available for one trait at $p$ SNPs (*e.g.,* Heart Atrial Appendage expression of SCN5A) while summary statistics for only the top SNP ${Z}_{2,obs}$ in a region is reported for the second trait (*e.g.* a SNP associated with QT interval from the GWAS Catalog). Like the original coloc method, we compute posterior probabilities of five mutually exclusive and exhaustive hypotheses:

- ${H}_{0}$: Neither trait has a causal SNP in the region
- ${H}_{1}$: Only trait 1 has a causal SNP in the region
- ${H}_{2}$: Only trait 2 has a causal SNP in the region
- ${H}_{3}$: Both traits have a causal SNP in the region, but the two causal SNPs are different
- ${H}_{4}$: Both traits have a causal SNP in the region, and the two causal SNPs are the same

of which, we are primarily interested in the posterior probability of ${H}_{4}$.

**Fig. 1:**
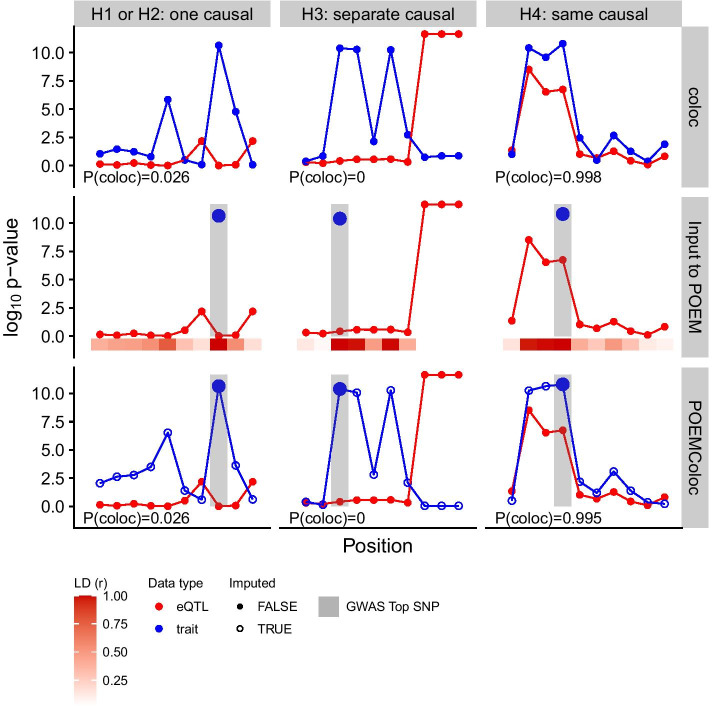
Illustration of the POEMColoc method compared to coloc. Under ${H}_{1}$ or ${H}_{2}$, only one of the two traits has any causal SNP in the region. Under ${H}_{3}$, the two traits have two different causal SNPs. Under ${H}_{4}$ (colocalization) the two traits share a causal SNP. The first row of the plot illustrates the coloc method using $p$-values. Input data is $p$-value at each SNP in the region for each trait, and the output is a posterior probability of each hypothesis. The posterior probability of colocalization (${H}_{4}$) is shown on the plot. The second row illustrates input to the POEMColoc method. Full summary statistics are available for one trait only, and for the second trait only the position and $p$-value of the top SNP is known. We also require LD from the top SNP to at least some of the trait 1 SNPs to be known from a reference panel (shown below in red). We use the LD to impute missing $p$-values for input to coloc. Imputed $p$-values are shown in the bottom row. POEMColoc consists of applying coloc to the imputed datasets and outputting posterior probabilities of colocalization (shown in the bottom row)

Our method first imputes missing summary statistics using both the summary statistics for the reported variant and the LD structure of the region in a reference population (Fig. 1). For a single causal SNP indexed by $c$, Z scores at the full set of SNPs for trait 2 (**Z**_**2**_) can be approximated by a multivariate normal distribution with parameters

$${\mathbf{Z}}_{2}\sim {\mathcal{N}_p}({\varvec{\upmu}},\Sigma )$$

where the covariance matrix is approximated by:

$${\Sigma }_{ij}={r}_{ij}$$

where ${r}_{ij}$ is the Pearson correlation of genotype dosages between SNPs $i$ and $j$.

$E({Z}_{i})$ is approximated by:

$${\mu }_{i}=\lambda {r}_{ic}$$

where ${r}_{ic}$ is the Pearson correlation between SNP $i$ and the causal SNP $c$ [8] and $\lambda$ is the standardized effect size at the causal SNP. A reasonable point estimate of the unknown parameters is $\lambda ={Z}_{2,obs}$ and $c=obs$. That is, we use the Z-score and index of the reported top SNP as a point estimate for parameters of the causal SNP (in fact, this estimate is a maximizer of the full data likelihood under the approximate normal distribution, Supplement Text S1). Using this point estimate of $\lambda$ and $c$, we can compute the expected value of the missing summary statistics conditional on the observed summary statistic at the top SNP. This conditional expectation formula has been used in previous work on the imputation of summary statistics at untyped SNPs [9, 10].

$$\tilde{Z}_{2,mis} = \mu_{mis|obs} = Z_{2,obs} r_{mis,obs}$$

Similar to coloc, POEMColoc accepts either $p$-values or coefficient estimates $\beta$ and $\mathrm{var}(\beta )$ as input. In practice, we expect $p$-values to be the most common form of input to POEMColoc as these are more commonly reported. We transform two-sided $p$-values to Z-scores using inverse cdf function $Z={F}^{-1}(p/2)$. We then apply $F$ to the imputed values $\tilde{Z}_{2} = \left( {\tilde{Z}_{2,1} , \ldots ,\tilde{Z}_{2,obs - 1} ,Z_{2,obs} ,\tilde{Z}_{2,obs + 1} , \ldots ,\tilde{Z}_{2,p} } \right)$ to obtain two-sided $p$-values $2F(-|Z|)$ and use these imputed $p$-values as input to *coloc.abf* from the *coloc R* package, along with $p$-values or $\beta$ values for dataset 1. In many cases, such as the UK Biobank association statistics we analyze in this paper, $p$-values are known to have been generated from a $t$-distribution with approximately $N$ degrees of freedom, but in the absence of other information we may also take $F$ to be the standard normal cdf (as does *coloc* with $p$-value input). Like the $p$-value implementation of coloc, POEMColoc additionally requires sample size and, for case–control studies, case fraction. Minor allele frequency and $r$ are either user-supplied or computed from a reference panel using SeqArray [11].

When neither of the datasets have full summary statistics, the imputation is performed for both datasets on all SNPs in a reference panel within a user-specified window of either top SNP, optionally subject to a minor allele frequency cutoff. When necessary, we will distinguish these two versions of coloc using POEMColoc-1(POEMColoc with one imputed dataset) and POEMColoc-2 (POEMColoc with two imputed datasets).

### POEMColoc performance using UK Biobank phenotypic associations and GTEx expression QTL

To assess the accuracy of our method at recovering the colocalization posterior probabilities using full summary statistics and the original coloc method, we used the combination of binary and quantitative trait associations from the UK Biobank (UKBB) and whole blood gene expression associations from the GTEx project [12]. In total, we obtained 646,884 combinations of UKBB phenotype associated window and GTEx eQTL summary statistics. We ran colocalization analysis on each of these using the original coloc method with full summary statistics. We find that approximately 0.4% of tested pairs are colocalized ($P({H}_{4})>0.9$).

**Fig. 2:**
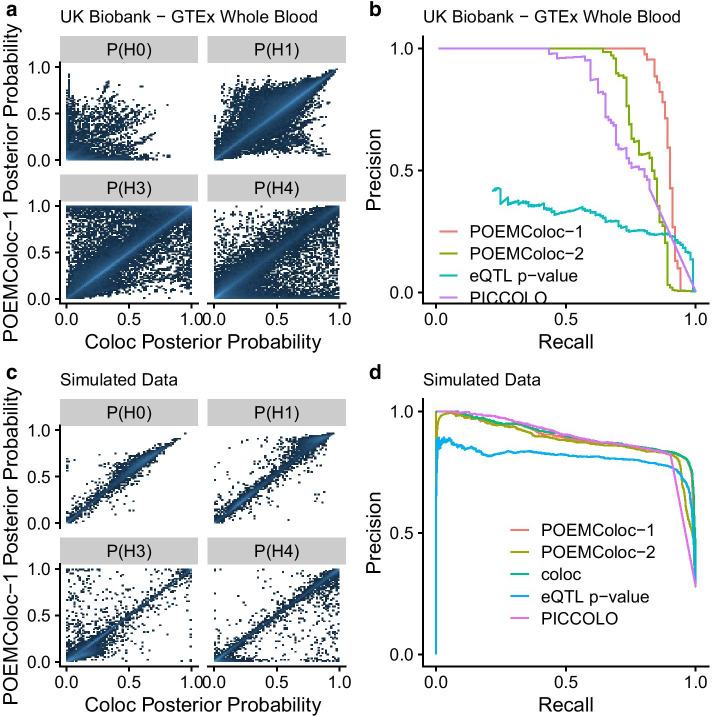
**a** Comparison of hypothesis posterior probability using POEMColoc-1 method to coloc using GTEx whole blood eQTL regression coefficients and UK Biobank summary statistics as input. **b** Precision-recall curves for predicting colocalization with GTEx whole blood eQTL using coloc ($P({H}_{4})>0.9$) in the UK Biobank. **c** Comparison of hypothesis posterior probability between POEMColoc-1 and coloc in simulated datasets. **d** Precision-recall curves for predicting colocalization (${H}_{4}$) in simulated datasets, compared to coloc and a simple method using the eQTL $p$-value of the GTEx top SNP

Next, we imagined the scenario in which instead of having full summary statistics for the UKBB phenotypes, these phenotypes had been reported in biomedical literature and only the most significantly associated variant for each locus was available. We used our method as implemented in the associated R package *POEMColoc*. Posterior probabilities of each hypothesis were highly correlated between POEMColoc and coloc (Fig. 2a, ${R}^{2}=0.81$ for $P({H}_{4})$). Use of $p$-values in place of regression coefficients as the input to coloc improves correlation for some hypotheses but has minimal effect on the posterior probability of colocalization ${H}_{4}$ (Additional file 1: Figure S1). Furthermore, treating colocalization as a binary classification problem where full coloc $P({H}_{4})>0.9$ was considered a true positive and all other outcomes were considered true negatives, we achieve a high degree of sensitivity and specificity using POEMColoc (AUCPR = 0.91 for POEMColoc-1 and 0.84 for POEMColoc-2). We performed colocalization analysis for a subset of phenotypes using PICCOLO, and found POEMColoc’s colocalization posterior probabilities more closely matched those of coloc, even when using only data from the top SNP from both traits, in datasets to which both methods could be applied (Fig. 2b, Additional file 1: Table S1, Figure S2). The large drop in POEMColoc precision at high values of recall seen in Fig. 2b is due to a minority of datasets with very high colocalization probability using coloc, but colocalization probability near zero using POEMColoc, and is discussed further in the section concerning multiple independent colocalizations in a region.

### Methods comparison for simulated associations

While our stated goal was to approximate coloc using full summary statistics, performance assessments using coloc as the gold standard cannot compare performance between coloc and POEMColoc at correctly assigning associations to the true hypothesis. To address these limitations we compared the performance of POEMColoc and coloc on simulated data and compared both to PICCOLO and to what performance would be if we used the simple approach of quantifying causal evidence using the eQTL $p$-value of the top GWAS SNP. Hypothesis posterior probabilities calculated with POEMColoc are highly correlated with posterior probability calculated with coloc method (Fig. 2c, ${R}^{2}=$ 0.98 for the posterior probability of colocalization). A major advantage of POEMColoc over PICCOLO is that because PICCOLO requires the top SNP in both datasets to be found in an old release of 1000 Genomes, POEMColoc can be run on a much larger fraction of simulated datasets (97% versus 39%). Considering simulated datasets to which both POEMColoc and PICCOLO could be applied, the precision recall plot in Fig. 2d shows that the performance of POEMColoc is close to the coloc method, better than the simple method (eQTL *p*-value) of using the eQTL $p$-value of the GWAS top SNP, and has higher AUCPR than PICCOLO, even when using only the top SNP from each dataset (Additional file 1: Table S1).

## POEMColoc caveats

### Effect of reference panel ancestry

**Fig. 3:**
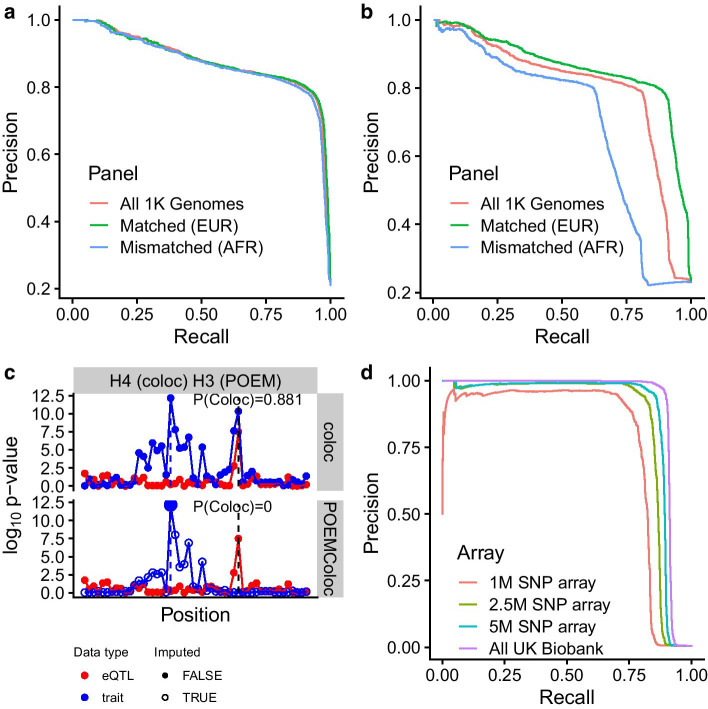
Scenarios that reduce POEMColoc performance. **a** POEMColoc-1 sensitivity to mismatched ancestry panels. Performance at identifying colocalization (${H}_{4}$) in datasets simulated under ${H}_{0}-{H}_{4}$. Data was simulated from European only ancestry individuals and the indicated population was used as a reference panel. **b** POEMColoc-2 sensitivity to mismatched ancestry panels. Performance at identifying colocalization (${H}_{4}$) in datasets simulated under ${H}_{0}-{H}_{4}$. **c** An example of a dataset in which there are multiple independent GWAS signals. Only the second peak is colocalized with the eQTL, so POEMColoc does not detect it. **d** Precision-recall curves for predicting colocalization using coloc ($P({H}_{4})>0.9$) in the UK Biobank where the POEMColoc top SNP was restricted to SNPs on different sized Illumina array genotyping subsets (1 M—OmniExpressExom8; 2.5 M—HumanOmni2.5Exome; 5 M—HumanOmni5Exome)

We next compared performance of POEMColoc when the ancestry of the reference panel was mismatched to the GWAS study ancestry. We can see that when GWAS data are simulated using British ancestry genotypes, performance of POEMColoc is better using individuals from the 1000 Genomes European superpopulation as a reference panel than when using all 1000 Genomes individuals or a mismatched superpopulation. Differences between ancestry panels are minor given that the GWAS top SNP exists in ancestry panel and when only one of the two datasets requires imputation (Fig. 3a). The largest difference comes from the proportion of top SNPs that can be found in the panel (and therefore the proportion of datasets to which POEMColoc can be applied). While only 3% of GWAS top SNPs from the simulation were missing from the 1000 Genomes European samples, 12% were missing from the 1000 Genomes African samples. When both datasets require imputation, effects are more substantial (Fig. 3b). Additionally, when the smaller eQTL dataset is imputed, performance is more sensitive to ancestry mismatch than when the larger GWAS dataset is imputed (Additional file 1: Figure S9). In simulation, mismatched ancestry generally reduces recall more than precision using a typical cutoff value of 0.9 (Additional file 1: Table S2) and lowers estimates of colocalization probability (Additional file 1: Figure S10). Overall, we find that a well-matched ancestry panel improves performance, but that the method will be usable, but conservative in the presence of mismatched ancestry.

### Multiple independent associations in region

While we find that across all tested regions, the correlation between colocalization probabilities is very high, there were notable exceptions. In particular, there is a population of tests which has high ($>0.9$) probability of colocalization using full summary statistics, but that has a low ($<0.1$) probability of colocalization using POEMColoc (a false negative population). This accounts for 9% of datasets with colocalization posterior probability greater than 0.9 using coloc with full summary statistics. For all false negative datasets, the highest posterior probability hypothesis according to POEMColoc is ${H}_{3}$ (two separate causal signals). Within the population of false negatives, we noticed many in which there appear to be two peaks. Figure 3c shows an illustrative example. The lower GWAS peak appears to have a colocalized eQTL peak, but the GWAS peak containing the top SNP does not appear colocalized. Indeed, false negative datasets are enriched for datasets with similar UKBB $p$-value between the GTEx top SNP and UKBB top SNP, but low LD between them, a scenario consistent with multiple independent UKBB causal SNPs (Additional file 1: Figure S15A). In this scenario, when we impute new association statistics from only the top UKBB SNP in the region, we only recover association statistics that were in LD with this top peak and which do not have a corresponding eQTL peak. The authors of coloc indicate that colocalization probability is generally based on the strongest association signal, but will be affected by multiple causal variants explaining a similar proportion of variance in the trait, and recommended performing colocalization conditional $p$-values [2]. We find performing a conditional analysis on the full summary statistics using GCTA-COJO [13] reduces colocalization discrepancies in many datasets (Additional file 1: Figure S15B). False negative datasets are more likely than true positive datasets to have multiple associations detected using COJO (Additional file 1: Table S3). More strikingly, in true positive datasets, for which POEMColoc and coloc agree on a high colocalization probability, switching to using conditional summary statistics rarely appreciably changes the probability of colocalization using coloc. However, for 43% of false negative datasets the colocalization probability changes by more than 0.5.

### Effect of GWAS study properties

GWAS studies reported in the biomedical literature use a variety of genotyping, sequencing, and imputation methods to obtain genome-wide genotypes on the study cohort. We reasoned that the performance of POEMColoc might depend on the accuracy and density of these genotypes as in turn, these details might affect the significance level and the probability that the top SNP was a good proxy for the causal SNP. The UKBB data presented above was derived from direct genotyping of approximately 850 K variants followed by imputation of approximately 90 M variants. A common alternative is for only directly genotyped variants to be tested. To simulate this scenario, we took our original analysis of full UKBB summary statistics and subset them as if one of 3 popular genotyping platforms spanning a range of genomic coverage had been used, now choosing only the top “directly genotyped” variant as the input to POEMColoc. We find that the POEMColoc method tends to miss colocalized signals rather than identify false colocalizations in this analysis and we find that performance suffers most when the set of directly genotyped SNPs is small (Fig. 3d, Additional file 1: Figure S4).

Another alternative is that genotypes are derived from whole genome sequencing WGS. This may be optimal for POEMColoc as we expect genotyping or imputation error would affect the ability of the top SNP to act as a proxy for the causal SNP. Additional file 1: Figure S6 shows a comparison between coloc and POEMColoc-1 for a selection of studies using WGS. The correlation of estimates of $\mathrm{P}({\mathrm{H}}_{4})$ between coloc and POEMColoc-1 was similar for WGS and imputed UKBB data (0.77 vs 0.81, respectively). Similarly, the proportion of false negatives ($\mathrm{P}({H}_{4})<0.1$ with POEMColoc-1 and $\mathrm{P}({H}_{4})>0.9$ with coloc) was comparable (9% in imputed vs 10% WGS). However, the total number of associations based on WGS is still small (174) relative to the UKBB and there are confounding factors like smaller sample size among WGS studies that make the interpretation of any differences difficult.

While we explored ancestry mismatch effects in simulations, we did not consider the effect of summary statistics from meta-analysis across diverse populations. We used a large such study to explore this effect analyzing data with $\sim$ 4000 hematological traits and from a sample size comparable to the UKBB. Again, compared to UKBB results, the correlation between $\mathrm{P}({H}_{4})$ of coloc and POEMColoc-1 was very similar (${R}^{2}=0.82$, Additional file 1: Figure S5) as was the proportion of false negatives (10%).

### Parameter sensitivity

The coloc prior allows SNP associations with the two traits to be non-independent. It has three parameters ${p}_{1}$, the prior probability a SNP is associated with trait 1, ${p}_{2}$, the prior probability a SNP is associated with trait 2, and ${p}_{12}$, the prior probability a SNP is associated with both traits. Higher values of prior parameter ${p}_{12}$ relative to ${p}_{1}{p}_{2}$ lead colocalization to be more favored over other hypotheses a priori. The default coloc prior may be too liberal [14]. Additional file 1: Figure S3 shows the correlation between POEMColoc-1 and coloc posterior probabilities is relatively insensitive to the value of ${p}_{12}$ in the UK Biobank, while using lower values of ${p}_{12}$ increases the correlation between POEMColoc-2 and coloc posterior probabilities.

We also considered how POEMColoc’s performance varied in simulations when heritability (Additional file 1: Figure S11) and sample size (Additional file 1: Figure S12) are reduced from the values used in Figs. 2 and 3. AUPRC decreases as heritability and sample size are reduced. When sample size in the imputed dataset(s) is sufficiently low, POEMColoc performance is substantially worse than coloc. Ancestry misspecification also has a greater impact when sample size is lower (Additional file 1: Figure S13).

GWAS sample size and heritability used in Figs. 2 and 3 were chosen so that the great majority of simulated datasets would contain a genome-wide significant result ($p<5\times {10}^{-8}$), as colocalization analysis is frequently performed to follow up on such genome-wide significant findings. Reducing either sample size or effect size leads to fewer genome-wide significant findings in simulated datasets. Binning all simulations under ${H}_{3}$ and ${H}_{4}$ by the lowest GWAS and eQTL $p$-values, we see that POEMColoc performance closely matches coloc when $p$-values are $<{10}^{-6}$ (Additional file 1: Figure S14). However, higher GWAS $p$-values will cause POEMColoc-1 (GWAS imputed) and POEMColoc-2 (GWAS and eQTL imputed) performance to degrade relative to coloc. Higher eQTL $p$-values have a large impact on the performance of POEMColoc-2 but not POEMColoc-1.

### Application to full GWAS catalog

**Fig. 4:**
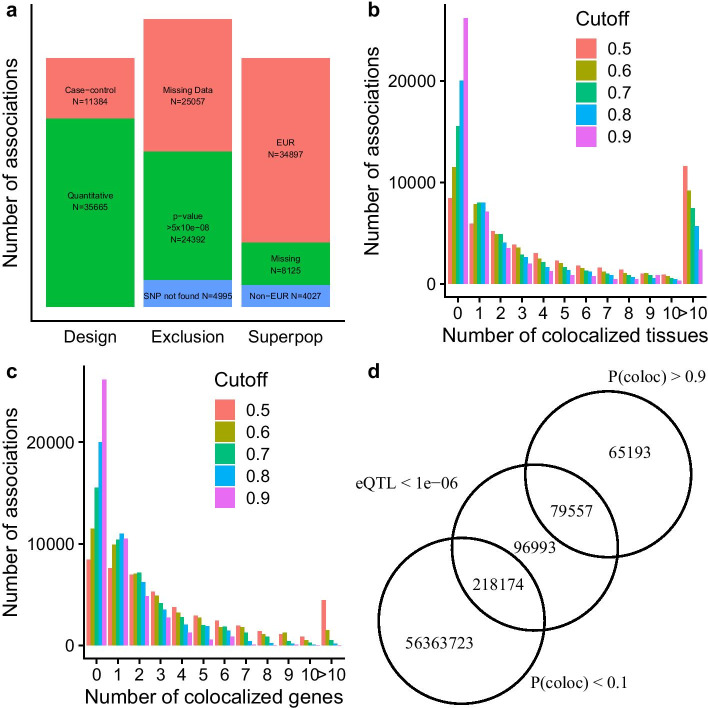
**a** Number of GWAS Catalog associations by design (left), reason for exclusion (middle), and superpopulation (right). **b** Number of colocalized tissues per GWAS Catalog association. **c** Number of colocalized genes per GWAS Catalog association. **d** Overlap between colocalized association-gene-tissue trios ($P({H}_{4})>0.9)$ and eQTL significance ($p<{10}^{-6}$) of the reported GWAS Catalog top SNP

Having confidence that the POEMColoc method accurately approximated the full coloc method in real data from UKBB and GWAS Catalog and that it recovered true colocalized signals generated from simulation, we sought to apply the POEMColoc method to GWAS results for which full summary statistics are not available. Figure 4a summarizes exclusion criteria, cohort ancestry, and study design among analyzed associations. In total, we assessed 47,049 reported GWAS associations with $p$-value $\le 5\times {10}^{-8}$ for colocalization. Note that associations excluded due to missing information were usually from difficult to parse sample size and design and in most cases should be possible to analyze using POEMColoc after manual review of the entry or linked PubMed article. Additional associations were excluded due to not being able to locate the associated SNP in our reference panel. Colocalizations with posterior probability greater than 0.9 are provided in Additional file 2: Supplementary dataset 1.

GWAS Catalog associations meeting inclusion criteria were tested for colocalization using GTEx eQTL in 48 tissues using the POEMColoc method. Our analysis detected 151,247 colocalized association-cis gene-tissue triplets, or 0.2% of those tested. The average run time of this analysis per triplet was 0.1 s. Although slower than coloc because of the need to compute LD from a reference panel, if multiple eQTL from a single locus are supplied, POEMColoc can take advantage of this to only compute LD once for the locus. Figure 4c shows the number of colocalized genes detected per association. Using cutoff 0.9, 44% of associations have at least one detected colocalization with a GTEx eQTL in some tissue, and 22% have colocalizations for more than one gene. Given there are one or more colocalized genes, the median number of tissues involved is 2, though much larger numbers of tissues are also common (Fig. 4b). More colocalizations are detected in tissues with larger sample sizes (Additional file 1: Figure S8). Performing colocalization analysis using POEMColoc yields substantially different results compared to using an eQTL $p$-value criterion (Fig. 4d). In fact, in most cases when the top GWAS SNP had a eQTL $p$-value less than ${10}^{-6}$ there was evidence against colocalization.

### Tissue enrichment

**Fig. 5:**
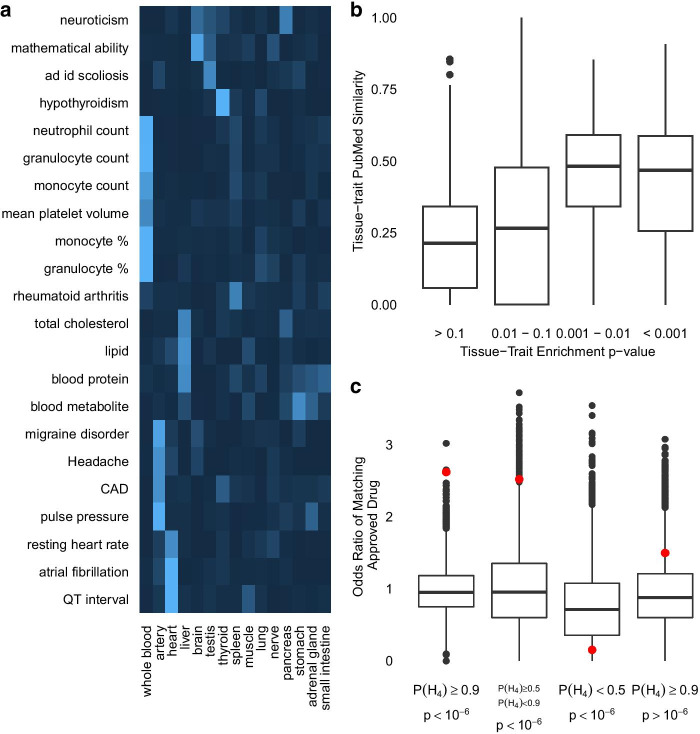
**a** Heat map of tissue enrichments for select traits with the largest enrichments. Brighter blue indicates more significant enrichment. CAD, coronary artery disease; ad id scoliosis, adolescent idiopathic scoliosis; granulocyte %, granulocyte percentage of myeloid white cells; monocyte %, monocyte percentage of leukocytes. **b** Average tissue-trait similarity for enriched pairs by enrichment $p$-value. Similarities are computed from cooccurance in PubMed articles. **c** Odds ratio of a gene-trait pair matching an approved drug mechanism by eQTL $p$-value of the GWAS top SNP and colocalization posterior probability P(${\mathrm{H}}_{4}$) (red dot). Odds ratios are computed relative to cis gene-trait pairs with both eQTL $p$-value $>{10}^{-6}$ and colocalization posterior probability less than 0.9. Boxplots show the distribution of this odds ratio using permuted GWAS trait labels

To evaluate the biological relevance of POEMColoc output, we assess whether colocalizations are enriched in disease-related tissues. Figure 5a shows tissue enrichments for traits with both large numbers of colocalizations and significant enrichment in some tissue (enrichment *p*-value $<0.001$ and at least 7 colocalized genes). For figure legibility, the 48 GTEx tissues have been collapsed into 27 tissue groups as provided by GTEx and the top 15 enriched tissue groups across the selected traits are shown. These enrichments are largely biologically interpretable, for example enrichment of blood count phenotypes in whole blood, lipid, cholesterol and protein measurements in the liver, and hypothyroidism in the thyroid. Additional file 1: Table S5 gives a complete list of enrichments detected at the tissue level. We also provide a comparison to enrichments that would be detected using eQTL $p$-values and show that some biologically interpretable enrichments (e.g. QT interval in the heart left ventricle) are only detected using POEMColoc. Because of the large-scale nature of this analysis and because we wish to reduce the impact of human biases in evaluating the method, we quantify disease-related tissues using an independent and automated approach that quantifies the co-occurrence of tissues and traits in the biomedical literature using a standardized MeSH vocabulary. Significantly enriched tissue-trait pairs using POEMColoc are more similar by the literature co-occurrence metric than those that are not enriched (Fig. 5b).

### Ability to predict approved drug mechanisms

We ask whether colocalized associations are enriched for matches to approved drug mechanisms, an independent source of evidence for target involvement in disease using methodology and supplementary datasets from [15] and [16]. Additional file 1: Table S6 shows matches to approved drug mechanisms from colocalized GWAS Catalog eQTL. We find significant enrichment for both significant top SNP eQTL and colocalized eQTL (Fig. 5c). The largest enrichment is found when there is both an eQTL significant at ${10}^{-6}$ and high to intermediate posterior colocalization probability. There are few matches to approved drug mechanisms for significant eQTL with low $P({H}_{4})(<0.5$). In part, this may be because these eQTL are less likely to be coding, and if coding, are less likely to be drug targets than those with high or intermediate colocalization posterior probability (Additional file 1: Figure S18). Upon examination of Additional file 1: Table S6 we see many closely related drug mechanisms are supported by colocalization evidence (e.g. PPARA for Hypercholesterolemia and Hyperlipidemias). In order to account for this, we counted the number of distinct targets with approved drug mechanisms, and find significantly higher counts than expected by chance (35 targets, $p<0.0001$).

## Discussion

We report a new method for performing colocalization analysis when only the summary statistics at a lead SNP are reported (POEMColoc). We show that the method provides a close approximation to the popular coloc method in application to real association summary statistics from the UK Biobank or GWAS Catalog and GTEx projects. Application to colocalization of associations from the GWAS Catalog and GTEx eQTL provides 22,673 distinct colocalization-based links between gene and traits with posterior probability 0.9 or higher. Applying colocalization analysis gave substantially different results than relying on an eQTL statistical significance criterion for the lead GWAS SNP.

When there are large differences between POEMColoc and coloc, most commonly coloc gives a high probability of colocalization and POEMColoc a low probability. The “false negative’’ population is relatively smaller in our simulated datasets, but occurs more often in mismatched ancestry panels (Additional file 1: Table S4) and under lower GWAS sample size (Additional file 1: Figure S16). In the UK Biobank analysis, we find that this “false negative” population is enriched for regions where there is evidence of multiple causal variants in the GWAS trait. When multiple causal variants are present, imputed summary statistics used by the POEMColoc method will only be accurate for variants linked to the top SNP. Recent changes to coloc (not part of the CRAN release as of writing), are designed to account for the scenario of multiple causal variants [14]. However, we do not expect these developments to apply to POEMColoc imputed datasets due to having summary statistics from only one variant. Comparing the size of the false negative population using POEMColoc-1, POEMColoc-2, and PICCOLO, we find it is largest using PICCOLO (9%, 15%, 27% of UK Biobank loci—whole blood eQTL pairs on which all three methods could be run and coloc using full data detected a positive result respectively). We find the greatest number of true positives using POEMColoc-1 and somewhat greater numbers of true positives detected using PICCOLO relative to POEMColoc-2, likely due to this method’s greater tendency to output probabilities close to zero and one (Additional file 1: Figure S17).

Our method uses a population reference panel to obtain LD estimates and from these, we impute missing summary statistics in the region. As LD can differ between populations of different ancestries, we wondered how the method would perform if there was a mismatch between reference panel ancestry and the original GWAS ancestry. Our simulations show that mismatched ancestry has a minor effect on POEMColoc-1 and while the best performance is achieved when GWAS and reference panel ancestries are matched, overall ability to correctly assign causal configurations in simulated data remains high when using completely mismatched ancestry panels (e.g., all European vs all African) provided that the GWAS study is well-powered. In real data, we see similar performance between GWAS datasets derived from imputed vs directly sequenced genotypes and similar performance comparing single-population studies to multi-ethnic meta-analysis. We observed a more substantial decay in performance of POEMColoc-2 and POEMColoc-1 when the top SNP reported in the GWAS catalog comes from a limited subset of directly typed variants. In simulations in which we vary sample size of the GWAS and eQTL datasets, we find reduced performance of POEMColoc-1 relative to coloc as GWAS sample size is reduced, and POEMColoc-2 as both eQTL and GWAS sample size are reduced, suggesting sample size in the imputed dataset has an important effect on performance (Additional file 1: Figure S12). The specific value of sample size required for POEMColoc performance to be comparable that of coloc will depend on trait heritability. For example, an eQTL sample size of 500 is adequate assuming a mean heritability of 0.1, but imputing summary statistics for a GWAS with sample size 2000 and mean heritability 0.0099 leads to a greater performance reduction. Subsetting simulated datasets by lead SNP *p*-value for GWAS and eQTL datasets, we find that when both GWAS and eQTL *p*-values are less than ${10}^{-6}$, POEMColoc performance tends to be close to that of coloc (Additional file 1: Figure S14).

Our approach deals with the specific scenario where only summary statistics for the top associated variant in a region is reported. However, one may encounter situations where summary statistics for a panel of variants are reported in a region. Here, it may be beneficial to use more sophisticated summary statistic imputation methods (e.g., [17]–[21]) and follow this imputation with colocalization analysis. Further, the POEMColoc approach does not account for uncertainty in linkage disequilibrium, the identity or strength of signal at the causal SNP in the imputed dataset, or in uncertainty in the imputed summary statistics. We expect that accounting for these could increase performance under some scenarios at additional computational cost. We show that colocalized gene-trait pairs are enriched in tissues relevant to the underlying GWAS trait. Some of these enrichments seem intuitive and confirm colocalizations inferred by the POEMColoc method can recover known relationships between traits and relevant tissues. Other trait-tissue enrichments are not as intuitive but are supported by known disease biology. Total cholesterol, low density lipoprotein, and lipid levels are enriched for eQTL colocalizations in the liver, a major site of both production and metabolism of cholesterol and LDL. Migraine and headaches are most enriched for colocalizations with artery eQTL and several lines of evidence including the ability of vasoactive substances to induce migraine, the effective treatment of migraines with drugs acting in the vascular system, and increased comorbidity of migraines and cardiovascular diseases support a causal role of the vascular system on migraine [22]. Overall, we find that significant enrichments in colocalizations between traits and tissues is predictive of the co-occurrence rates of the same traits and tissues in the PubMed literature. Drug targets with genetic support for their therapeutic hypothesis are more likely to result in approvals than drug targets without genetic support [15, 16]. We recently demonstrated that this increase in approval probability seems to depend on the type and quality of genetic support with genetic support derived from Mendelian associations and GWAS associations with a clear causal gene having a stronger effect than GWAS associations where the causal gene was more uncertain [16]. We tested whether colocalized gene-trait pairs were enriched among approved target-indication pairs. Indeed, we find that gene-trait pairs that have evidence of colocalization and evidence of an eQTL association at the trait associated locus are enriched by approximately threefold in approved target-indication pairs. This enrichment is not found in gene-trait pairs that have evidence of an eQTL at the trait associated locus but do not show evidence of colocalization with the trait. Thus, evidence of colocalization from the POEMColoc method may be useful for identifying drug targets that are more likely to succeed in clinical development.

## Conclusions

We report a new method for performing colocalization analysis when only the summary statistics at a lead SNP are reported. We show that the method provides a close approximation to the popular coloc method in simulations and in applications to real association summary statistics from the UK Biobank and GTEx projects. We find that our method performs well even when reference panels are not perfectly matched to study populations. We find significant reduction relative to coloc in performance where there are likely multiple causal variants, when reported top summary statistics come from a limited subset of genomic variation (e.g., only variants typed on a low density SNP-chip), and under conditions of low heritability and sample size in the imputed dataset. By imputing information, POEMColoc allows for the statistically principled testing of competing hypotheses of shared causality where previously not possible.

## Methods

### Evaluating POEMColoc on GWAS hits and GTEx eQTL

Full summary statistics for GWAS conducted on UKBB phenotypes were downloaded on 4 June 2019 from http://www.nealelab.is/uk-biobank/*.* We removed duplicated phenotypes by excluding sex-specific GWAS runs and excluding raw quantitative variables in favor of the inverse rank transformed alternative. For each GWAS, we selected non-overlapping windows around genome-wide significant ($p<5\times {10}^{-8}$) lead SNPs as candidates for colocalization with eQTL. Variants marked as low confidence were excluded (defined by minor allele frequency $<0.001$ and additionally for case–control $<\frac{25}{2{n}_{case}}$). Starting with the most significant SNP for a given trait, we extracted summary statistics in a 4 Mb window surrounding the lead SNP. Now excluding the top region, we chose the next most significant lead SNP and extracted all summary statistics in 4 Mb window. We stopped this process when no more genome-wide significant lead SNPs existed for that GWAS. For each UKBB window described above, we selected eQTL summary statistics from GTEx release 7 whole blood eQTL of the entire cis-candidate window of any gene containing associated UKBB variant within its cis-candidate window [12]. From the 2197 UKBB phenotypes with at least one genome-wide significant hit, there were 1553 phenotypes where at least one of the genome-wide significant hits overlapped a tested eQTL in GTEx whole blood. We merged summary statistics for all variants in common between the GTEx eQTL tests and the UKBB phenotype tests and used each gene, UKBB phenotype, and UKBB selected region as a way to evaluate POEMColoc in comparison to coloc. For each combination of UKBB and GTEx summary statistics, we applied coloc via the *coloc.abf* function from the *coloc R* package (version 3.2-1) using default priors. On the same combinations of UKBB and GTEx summary statistics, we ran POEMColoc using the full list of $p$-values from GTEx eQTL but using only the top SNP from the UKBB region as if it was reported in the GWAS catalog. We used LD information from unrelated European individuals in the 1000 Genomes phase3 reference panel as processed/distributed for use with the BEAGLE imputation software package (http://bochet.gcc.biostat.washington.edu/beagle/1000_Genomes_phase3_v5a/). We evaluated performance of POEMColoc under the scenario where the reported GWAS SNP came from a limited SNP subset by choosing the UKBB top SNP from only those SNPs contained on each of three Illumina genotyping chips that vary in total SNP content from 1 to 5 million SNPs (1 M—OmniExpressExom8; 2.5 M—HumanOmni2.5Exome; 5 M—HumanOmni5Exome).

To evaluate the performance of POEMColoc on a wider variety of GWAS types, we obtained summary statistics from the GWAS Catalog. To evaluate the effect of diverse ancestry, we obtained statistics for a single study of 15 hematological traits from meta-analysis of five global populations [23]. To evaluate the effect of direct sequencing vs genotype imputation, we obtained statistics from two additional studies reporting WGS derived summary statistics [24, 25]. We selected significant regions, merged with GTEx Whole Blood summary statistics, ran coloc and POEMColoc as described above for the UKBB summary statistics.

### Evaluating POEMColoc performance with simulations

Simulation studies were performed using genotype data from UK Biobank British ancestry individuals and simulated quantitative trait phenotypes. The LD structure in our simulation therefore reflects that of the UK Biobank British population. For each simulated dataset, a causal eQTL SNP was randomly selected among variants across the genome with minor allele frequency at least 0.01. Under *H*_*3*_, a second, distinct causal SNP was randomly selected from variants with minor allele frequency at least 0.01 within a 1 kb window from the first causal SNP. Close proximity of the two causal SNPs ensures that many datasets will present a challenging colocalization problem, in which the causal GWAS SNP may have a statistically significant association without shared causality due to linkage disequilibrium. We randomly sampled ${N}_{1}=500$ UK Biobank British individuals to simulate eQTL phenotypes (eQTL group), and ${N}_{2}=10000$ new individuals from the same population to simulate GWAS phenotypes (GWAS group). The phenotype for $i$th individual in group $k$ (${Y}_{ki}$) (where *k* = 1 indicates the eQTL dataset and *k* = 2 the GWAS dataset) is simulated based on the causal SNP genotype ${g}_{c}$ from UKBB data, ${Y}_{ki}\sim N({\beta }_{c}{g}_{ci},1)$ where ${\beta }_{c}=\sqrt{{h}^{2}}/\sqrt{2{f}_{c}(1-{f}_{c})(1-{h}^{2})}$ is computed from the minor allele frequency ${f}_{c}$ at site $c$ and single SNP heritability ${h}^{2}$. ${h}^{2}$ values were sampled from beta distributions with means 0.0099 for GWAS and 0.1 for eQTL (Beta(4, 400) and Beta(2, 18) respectively). We computed summary statistics for all SNPs with minor allele frequency greater than 0.01 within a 0.5 Mb window on either side of the causal eQTL variant. We compared POEMColoc with coloc, PICCOLO and with the simple approach using top SNP eQTL $p$-values. As in other analyses, coloc and POEMColoc were run using the default coloc priors on causal configurations. The analysis was run over the full 1 Mb region for which summary statistics were computed. Results from additional simulations using reduced heritability, ${N}_{1}$, and ${N}_{2}$ are presented in the supplementary materials.

### Comparison to PICCOLO

POEMColoc was compared to PICCOLO using its default settings and PICS causal probabilities obtained from *pics.download* using the EUR ancestry option. We attempted to run PICCOLO on all simulated datasets and a random sample of 100 UK Biobank phenotypes with at least one genome-wide significant association. Due to its use of PICS for estimating causal SNP probabilities and the fact that PICS uses only variants in 1000 Genomes phase I, PICCOLO is not able to run on a large fraction of analyzed datasets. All comparisons presented between PICCOLO and POEMColoc are restricted to the subset of datasets on which both could be run.

### Running POEMColoc on GWAS Catalog

GWAS Catalog data were downloaded on Nov 26, 2018. For each GWAS Catalog entry, we attempted to obtain the SNP $p$-value, whether the study is case–control or quantitative trait, the sample size $N$, and, for case–control studies, the fraction of observations that are cases. We also attempted to assign each entry to a broad ancestry group in order to choose an appropriate reference panel for imputation (broad ancestry groups matched 1000 Genomes superpopulations and were one of African, admixed American, East Asian, European, or South Asian). We excluded associations for which case–control or quantitative trait status and sample size could not be ascertained using our automated approach, and those not meeting the $p$-value threshold $5\times {10}^{-8}$. For each GWAS Catalog SNP rsid, we extracted GTEx summary statistics for all genes and tissues with available summary statistics overlapping the GWAS SNP. When a matched ancestry panel was available, POEMColoc was implemented using both the matched ancestry panel and the full 1000 Genomes from all unrelated individuals; otherwise the full 1000 Genomes was used. Using those associations for which a matched reference panel was available, we show that more colocalizations were detected using a matched reference (Additional file 1: Figure S7). For subsequent analyses, we used colocalization results from the matched ancestry panel where available and otherwise used the full 1000 Genomes. We evaluated the biological relevance of GWAS Catalog colocalizations using two different metrics, tissue enrichments of eQTL signals, and enrichments for approved drug mechanisms.

### Estimating tissue enrichment of eQTL signal

We collapsed replicate associations to obtain one colocalization posterior probability per gene-trait-tissue trio as the maximum across associations. Using a colocalization cutoff of 0.9, we determined an enrichment score for each trait-tissue combination as the $-{\mathrm{log}}_{10}p$-value from Fisher’s exact test for enrichment of colocalizations in the tissue-trait pair (Supplement Text S2).

### Association with approved drug targets

We used target-indication pair approval status from the supplementary materials of [16] to assess whether colocalization was associated with approval. xMHC targets (Chromosome 6 25.7–33.4 Mb) are unavailable because they were excluded from this analysis. Colocalizations were collapsed at the level of gene-trait pair by taking the maximum probability across tissues and genes. For each gene-trait pair assessed for colocalization, we determined whether or not it matched an approved drug mechanism. eQTL evidence classes were determined strong evidence for colocalization ($P({H}_{4})\ge 0.9$) evidence against colocalization ($P({H}_{4})\le 0.5$) and significant eQTL for the GWAS top SNP ($p<{10}^{-6}$), and combinations thereof. We computed the odds ratio of such a match for different positive classes of eQTL evidence relative to candidate pairs with no evidence of an eQTL via colocalization or eQTL $p$-value. It was determined that different evidence classes differed systematically in the proportion of coding genes and observed drug targets, so we conditioned this analysis on the candidate gene being an approved drug target. Significance was assessed via permutation of GWAS trait labels, which further helps separate ubiquitous target-level variation in colocalization and eQTL probability from the effect of the match between the target and the indication.

### PubMed odds ratio similarity

In order to identify drug mechanisms supported by colocalizations and to have a quantitative assessment of tissue-trait similarity, we used similarity in the MeSH vocabulary. Similarity is determined through odds ratio of cooccurance in article MeSH terms in the PubMed corpus (accessed October 10, 2018), an approach based on [26]. For identifying similar drug mechanisms, we used an odds ratio cutoff of 20 as this corresponded on average to the similarity cutoff used in the previous work. Drug indications are provided as MeSH terms in the supplementary materials of [16] and GWAS traits and tissues were mapped to the MeSH vocabulary using a similar procedure. Because of concerns about circularity from, for example, an approved drug leading to occurrence of its target and indication in publication, we exclude MeSH terms under the headings Amino Acids, Peptides, Proteins, Enzymes and Coenzymes, and Genetic Phenomena as well as all studies related to drug response or adverse reactions from the set of GWAS traits analyzed.

## Supplementary Information

**Additional file 1**. Supplementary methods and results.

**Additional file 2**. Supplementary dataset 1: A table of all high probability colocalizations between GWAS catalog entries and GTEX eQTL.

## Data Availability

POEMColoc R package is available at https://github.com/AbbVie-ComputationalGenomics/POEMColoc.

## Abbreviations

GWAS: Genome Wide Association Study
POEMColoc: Point Estimation of Colocalization
eQTL: Expression Quantitative Trait Locus
SNP: Single Nucleotide Polymorphism
RTC: Regulatory Trait Concordance
LD: Linkage Disequilibrium
UKBB: UK Biobank
AUCPR: Area Under the Precision-Recall Curve
MeSH: Medical Subject Headings
xMHC: EXtended Major Histocompatibility Complex
