# Supplementary material for: Estimating colocalization probability from limited summary statistics
Source: BMC Bioinformatics. 2021 May 17;22:254. doi: 10.1186/s12859-021-04170-z (PMC8130535; doi:10.1186/s12859-021-04170-z)
Supplement: Supplementary file 1 — Additional file 1. Supplementary methods and results. [file 12859_2021_4170_MOESM1_ESM.pdf]

S1 POEMColoc maximizes likelihood under approximate normal distribution

Assume a region with p SNPs. Assume a single causal SNP and let c be its index. Let λ be the expected value of the Z statistic at the causal SNP. The log likelihood under the approximate normal distribution is

$$l(\lambda, c|Z) = \frac{1}{2}\log|2\pi\Sigma| - \frac{1}{2}(Z - \lambda r_c)' \Sigma^{-1} (Z - \lambda r_c) \tag{1}$$

Absorbing constant terms with respect to the parameters

$$l(\lambda, c|Z) = \lambda r_c' \Sigma^{-1} Z - \frac{1}{2}\lambda^2 r_c' \Sigma^{-1} r_c = \lambda Z_c - \frac{1}{2}\lambda^2 \tag{2}$$

The simplification is due to r_c being a row of Σ. This is maximized at $\lambda = Z_c$ and $c = \text{argmax} Z_i$. The maximizing choice of c may not be unique, and the model may not be identifiable if two markers have $r = 1$.

Using these plugin estimates for c and λ we can impute $Z_{2,mis}$ with its conditional expectation given Z_{obs}.

$$\mu_{mis|obs} = Z_{obs} r_{mis,obs} \tag{3}$$

S2 Tissue enrichment computations

Let C_{ijk} be 1 if trait i is found to be colocalized with an eQTL of gene j in tissue k and 0 otherwise. We compute an enrichment odds ratio for tissue I and trait K and associated p-value from Fisher's exact test using the following 2×2 table.

$$\begin{array}{cc} \sum_j C_{IjK} & \sum_j \sum_{k \neq K} C_{Ijk} \\ \sum_j \sum_{i \neq I} C_{ijK} & \sum_j \sum_{k \neq K} \sum_{i \neq I} C_{ijk} \end{array}$$

Conditioning on colocalization when performing the enrichment analysis serves to mitigate the effect of confounding variables such as different colocalization probabilities across genes, traits and tissues. For example, there is a strong correlation between the tissue sample size and number of colocalizations detected (Figure S8). To motivate this computation consider the following model

$$p(C_{i,j,k} = 1) = \mu \alpha_i \beta_j \gamma_k \delta_{ik} \eta_{ij} \tau_{jk}$$

If $\delta_{ik} = 1$ for all i, k and $\tau_{j,k} = 1$ (no interactions between colocalization in tissue and colocalization in trait, and no interactions between colocalization in gene and colocalization in trait) for all j, k, expected cell counts are

$$\begin{array}{cc} \mu \alpha_I \gamma_K \sum_j \beta_j \eta_{Ij} & \mu \alpha_I \sum_j \beta_j \eta_{Ij} \sum_{k \neq K} \gamma_k \\ \mu \gamma_K \sum_{i \neq I} \sum_j \beta_j \alpha_i \eta_{ij} & \mu \sum_{i \neq I} \sum_j \beta_j \alpha_i \eta_{ij} \sum_{k \neq K} \gamma_k \end{array}$$

leading asymptotically to an odds ratio of

$$\frac{\mu\alpha_I\gamma_K\sum_j\beta_j\eta_{Ij}\mu\sum_{i\neq I}\sum_j\beta_j\alpha_i\eta_{ij}\sum_{k\neq K}\gamma_k}{\mu\alpha_I\sum_j\beta_j\eta_{Ij}\sum_{k\neq K}\gamma_k\mu\gamma_K\sum_{i\neq I}\sum_j\beta_j\alpha_i\eta_{ij}}=1$$

S3 Follow up on UK Biobank analysis using GTCA-COJO

The most common difference between POEMColoc and coloc applied to the UK Biobank was that a minority of datasets with high estimated colocalization probability using coloc applied to full summary statistics had low estimated colocalization probability using POEMColoc. We defined a "false negative" colocalization to be one in which coloc estimated the posterior probability of colocalization greater than 0.9, but POEMColoc estimated the posterior probability of colocalization be less than 0.1 and subjected them to further investigation. When looking at datasets "false negative" colocalization using POEMColoc, many appeared to have two separate peaks in the full dataset, but only one peak in the imputed dataset (Figure 3C). One of the peaks was colocalized with the eQTL, but it was not the highest. To address this, we ran GCTA-COJO [1] on the full summary statistics from UK Biobank to detect multiple causal SNPs within a 2 Mb window surrounding each top SNP from the UK Biobank using a p-value cutoff of 5×10^{-8} and colinearity parameter 0.8. A subsample of 5,000 individuals from the UK Biobank was used as a reference panel. When multiple SNPs were detected, we ran a conditional analysis for the top SNP conditional on all other SNPs. In some cases, the top SNP was not selected in the conditional analysis. If a SNP with LD at least 0.9 to it was, we used this in its place, otherwise the dataset was excluded from the conditional colocalization analysis. We recomputed colocalization probabilities using the conditional summary statistics as input to POEMColoc in place of the raw summary statistics. As a point of comparison, we also looked at how the same analysis changes the results for true positive or true negative datasets (defined by both POEMColoc and coloc having colocalization probabilities greater than 0.9 and less than 0.1 respectively).

S4 Precision-recall curve confidence intervals

Area under the precision-recall curve (AUCPR) was computed using the `precrec` `R` package [2]. Bootstrap confidence intervals were generated using `boot` `R` [3] for individual methods and pairwise differences in AUCPR. Following recommendations [4], we used a stratified bootstrap.

Table S1: Performance of methods in predicting coloc at cutoff 0.9 in the UK Biobank and predicting causal causal variants in simulation. Area under precision recall curve (AUCPR) and 95% bootstrap confidence interval. All confidence intervals for pairwise differences in AUCPR exclude zero (from bootstrap resampling of pairwise differences), except for the difference between PICCOLO and POEMColoc-2 on simulated datasets. % missing gives the percent of datasets that were not analyzed due to missing SNP information.

method	AUCPR UKBB	AUCPR Simulation	Missing % UKBB	Missing % Simulation
POEMColoc-1	0.9 (0.84,0.95)	0.9 (0.89,0.91)	5.3	3.3
PICCOLO	0.74 (0.66,0.82)	0.87 (0.86,0.89)	26.2	60.9
eQTL p-value	0.32 (0.25,0.38)	0.81 (0.79,0.82)	NA	NA
POEMColoc-2	0.81 (0.74,0.88)	0.88 (0.87,0.89)	5.9	6.6
coloc	NA	0.9 (0.89,0.91)	NA	NA

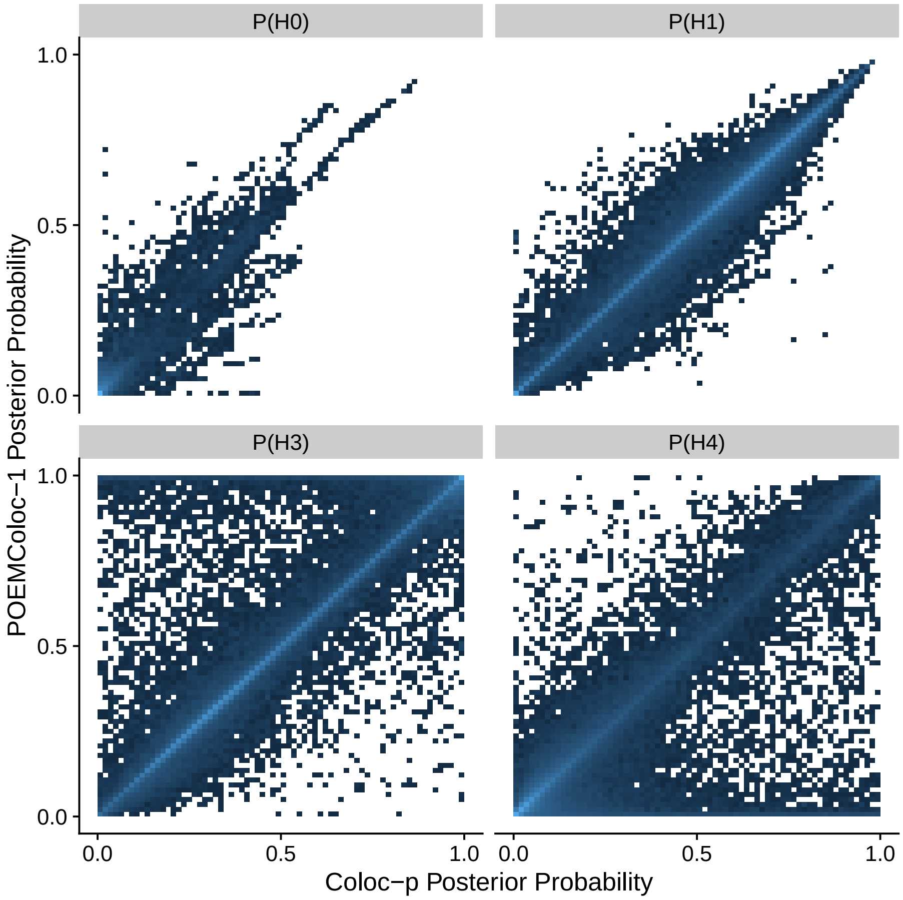

Figure S1: Comparison of hypothesis posterior probability ~~estimates~~ using POEMColoc-1 method to coloc using GTEx eQTL regression coefficients and UK Biobank p-value summary statistics as input (coloc-p)

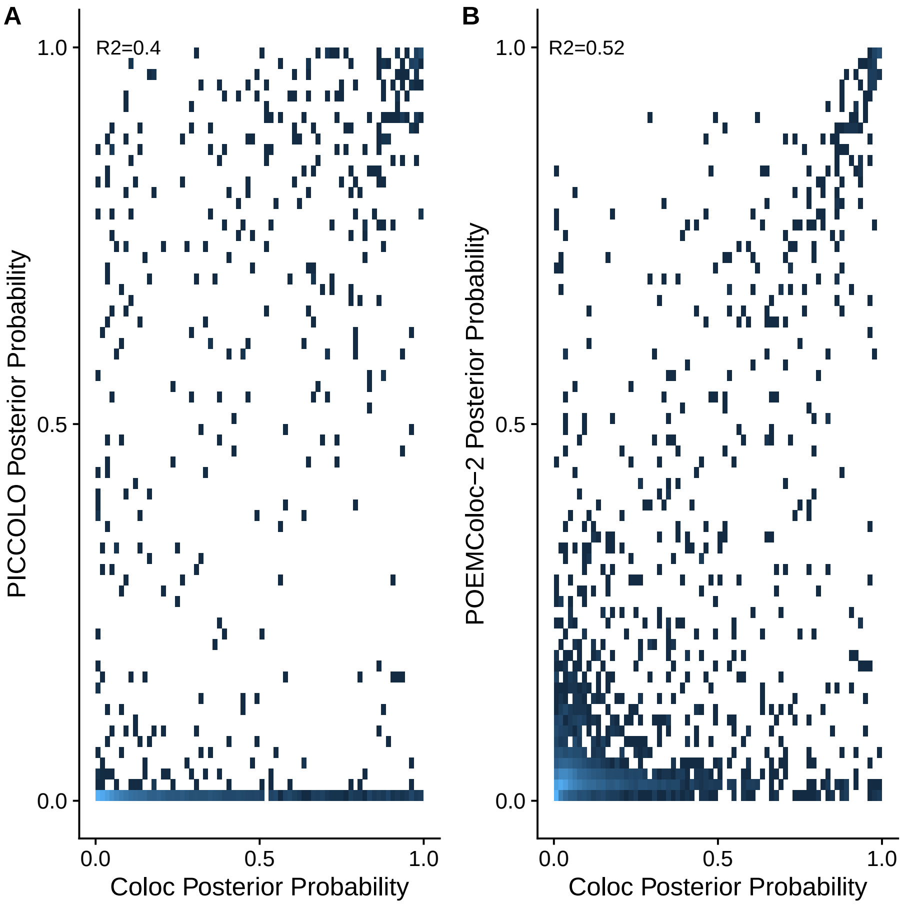

Figure S2: A: Comparison of colocalization posterior probability ~~estimates~~ between PICCOLO and coloc for GTEx eQTL and 100 UK Biobank phenotypes. B: Comparison of colocalization posterior probability ~~estimates~~ between POEMColoc-2 and coloc for GTEx eQTL and the same 100 UK Biobank phenotypes.

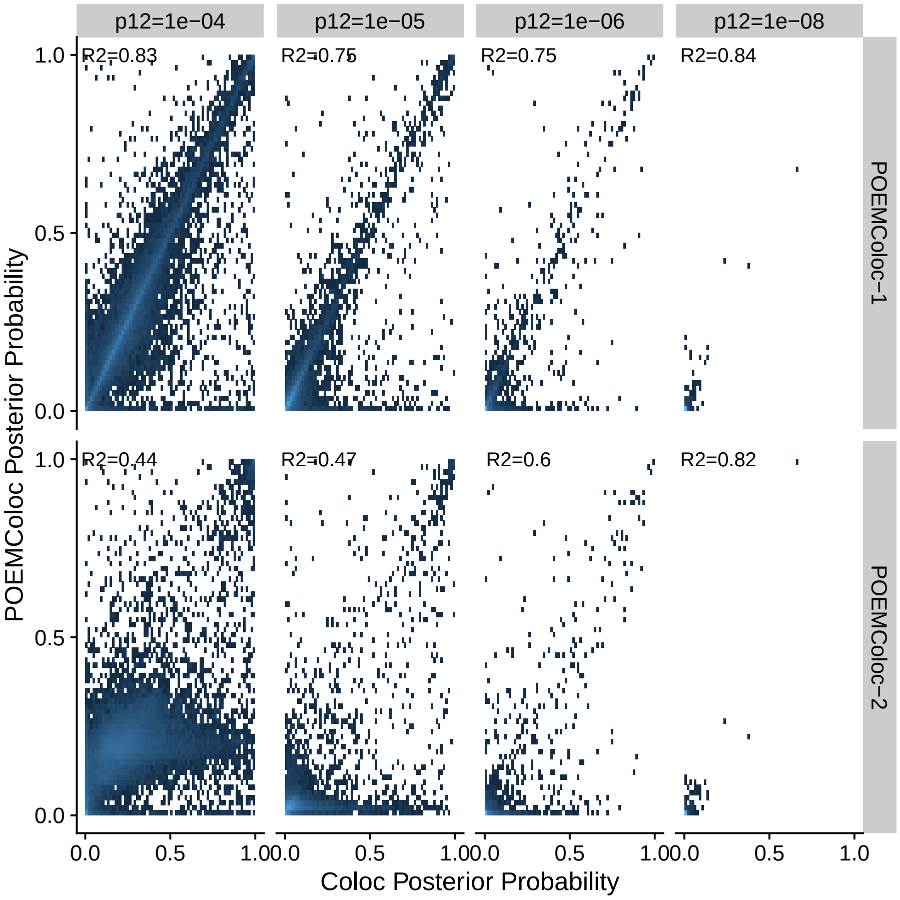

Figure S3: POEMColoc-1 (top) and POEMColoc-2 (bottom) colocalization posterior probability ($P(H_4)$) versus coloc posterior probability on the UK Biobank and GTEx whole blood eQTL. Comparison of $P(H_4)$ under different prior values of a SNP being associated with both traits (p_{12}). Prior probability of a SNP associated with a single trait is set to $p_1 = p_2 = 10^{-4}$. The default value from the `coloc` R package used elsewhere in this work is $p_{12} = 10^{-5}$, but $p_{12} = 10^{-6}$ is another common choice [5][6]. Labels show squared correlation R^2 between POEMColoc and coloc estimates of $Pr(H_4)$. Analysis uses a subset of 507 phenotypes.

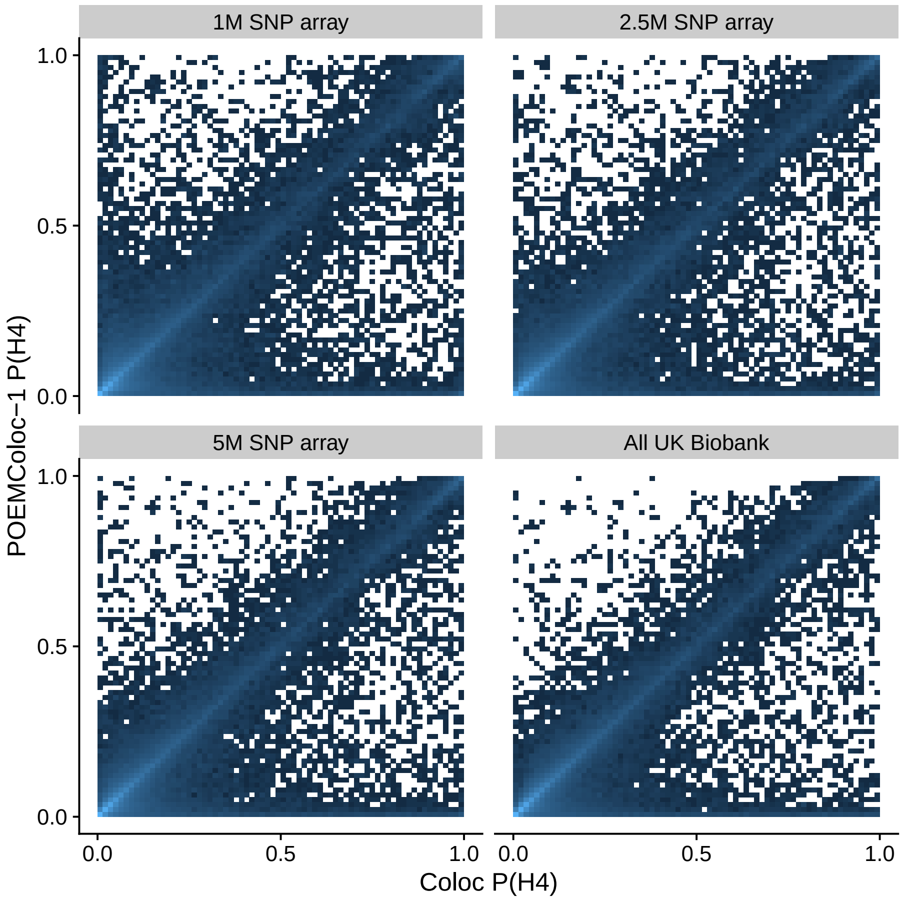

Figure S4: POEMColoc-1 colocalization posterior probability versus colocalization probability ~~estimated~~ using coloc on the UK Biobank and GTEx whole blood eQTL. Comparison of hypothesis posterior probability ~~estimates~~ for coloc and POEMColoc-1 where the POEMColoc top SNP was restricted to SNPs on different sized Illumina array genotyping subsets (1M - OmniExpressExom8; 2.5M - HumanOmni2.5Exome; 5M - HumanOmni5Exome)

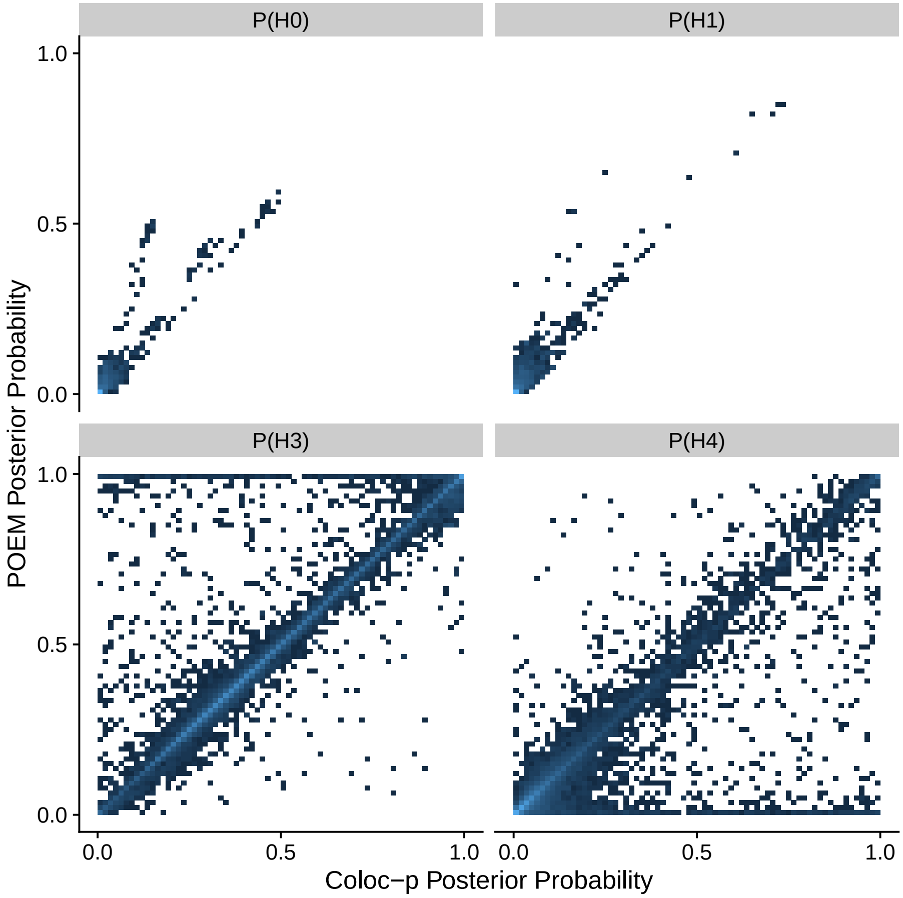

Figure S5: POEMColoc-1 colocalization posterior probability versus colocalization probability using coloc on GWAS summary statistics derived from meta-analysis of diverse populations and GTEx whole blood eQTL.

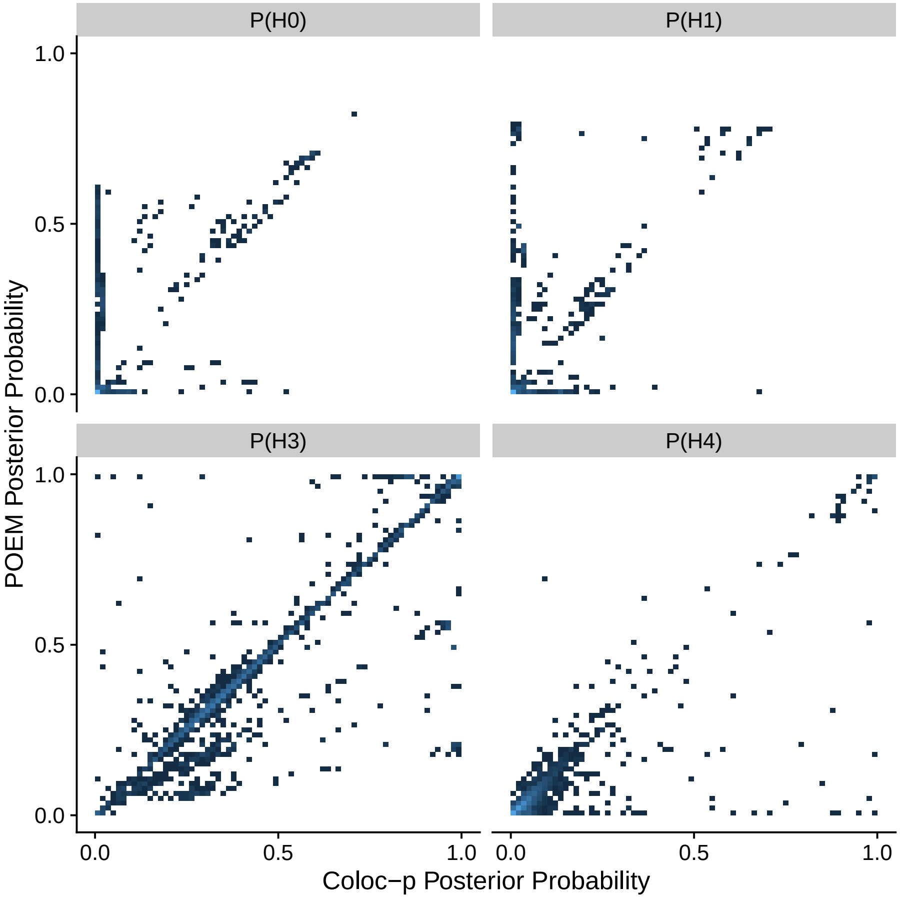

Figure S6: POEMColoc-1 colocalization posterior probability versus colocalization probability using coloc on GWAS summary statistics derived Whole Genome Sequencing and GTEx whole blood eQTL.

Table S2: Sensitivity of POEMColoc performance to ancestry panel misspecification in simulation study. Genotypes from UK Biobank European ancestry were used in the simulation, so 1000 genomes EUR is expected to be the best-matching panel. Missing % is the percent of simulated datasets that could not be analyzed due to the top SNP not being in the panel. Recall and precision are computed using a cutoff value of 0.9.

Method	Panel	AUCPR	Missing %	Recall	Precision
POEMColoc-1	EUR	0.88 (0.87,0.89)	3.3	0.68	0.83
POEMColoc-1	AFR	0.88 (0.87,0.89)	12.4	0.66	0.84
POEMColoc-1	All 1K Genomes	0.88 (0.87,0.89)	3.2	0.67	0.84
POEMColoc-1	AMR	0.88 (0.87,0.89)	4.1	0.67	0.83
POEMColoc-1	SAS	0.88 (0.87,0.89)	7.3	0.68	0.83
POEMColoc-1	EAS	0.86 (0.85,0.87)	28.9	0.65	0.82
POEMColoc-2	EUR	0.86 (0.85,0.87)	6.6	0.67	0.83
POEMColoc-2	AFR	0.72 (0.7,0.73)	22.5	0.36	0.85
POEMColoc-2	All 1K Genomes	0.81 (0.8,0.83)	6.3	0.50	0.85
POEMColoc-2	AMR	0.84 (0.83,0.85)	8.1	0.59	0.84
POEMColoc-2	SAS	0.84 (0.82,0.85)	13.7	0.61	0.83
POEMColoc-2	EAS	0.8 (0.78,0.81)	47.0	0.51	0.82

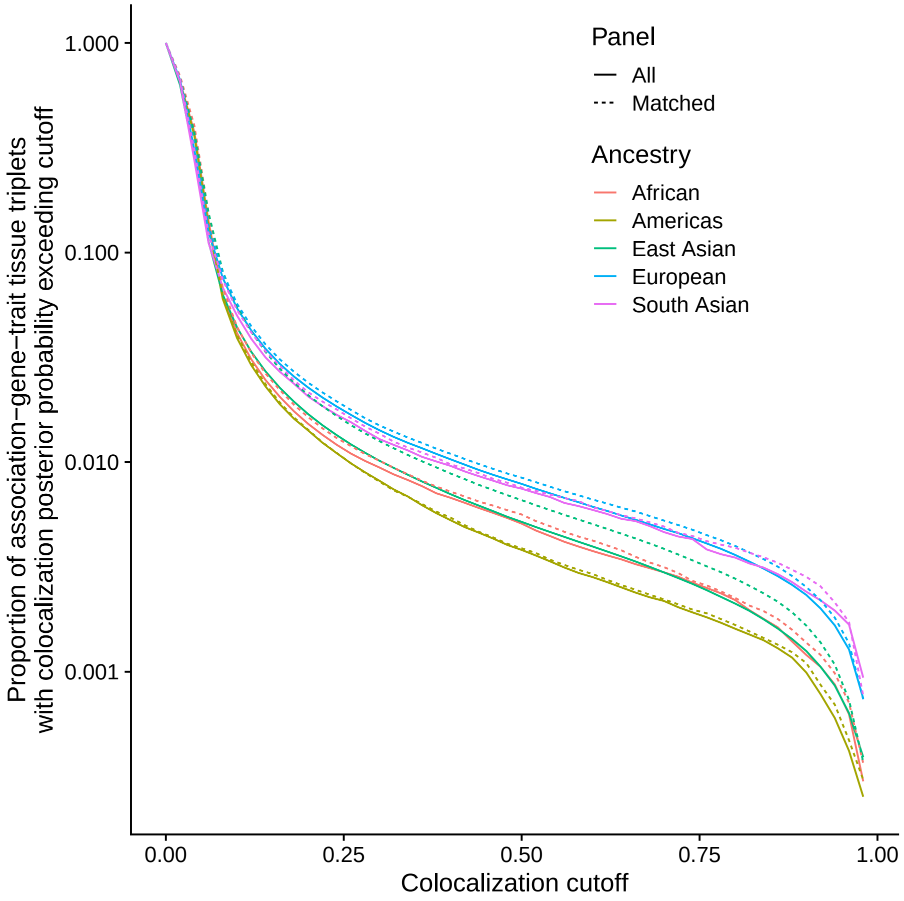

Figure S7: Proportion of association-gene-trait tissue triplet showing evidence for colocalization by colocalization cutoff. Colors correspond to study ancestry and solid and dashed lines to whether the imputation panel is matched ancestry or all 1000 Genomes related individuals.

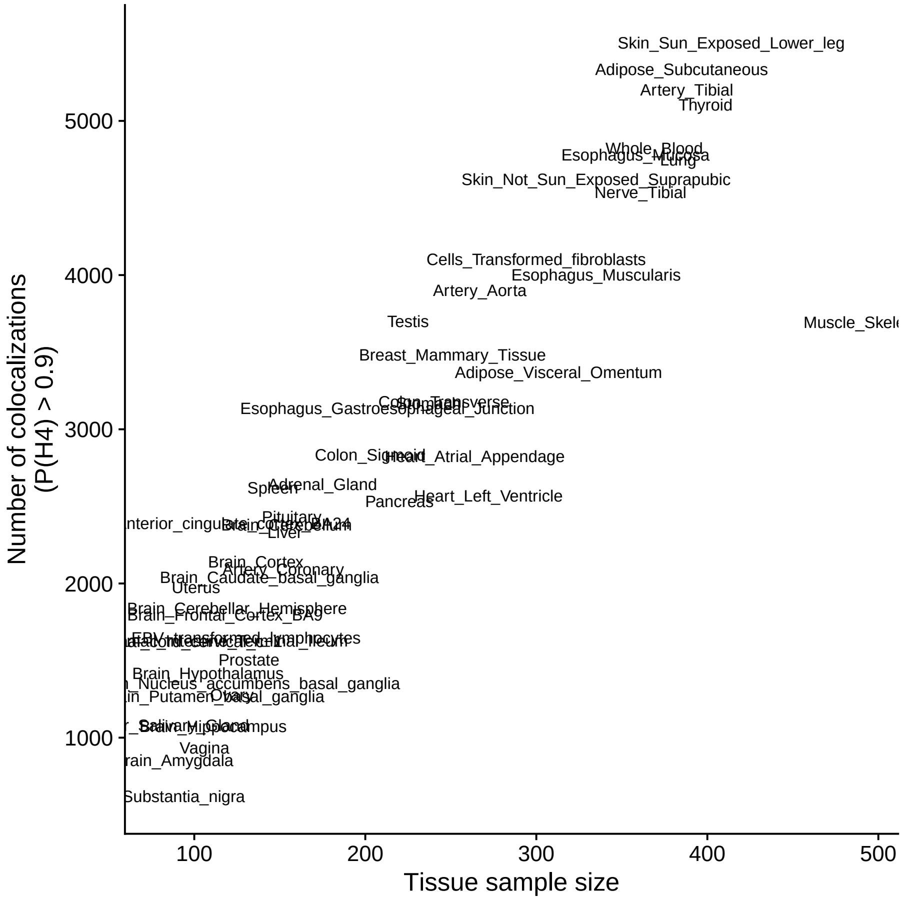

Figure S8: Number of colocalized association-gene pairs ($P(H_4) > 0.9$) by tissue sample size.

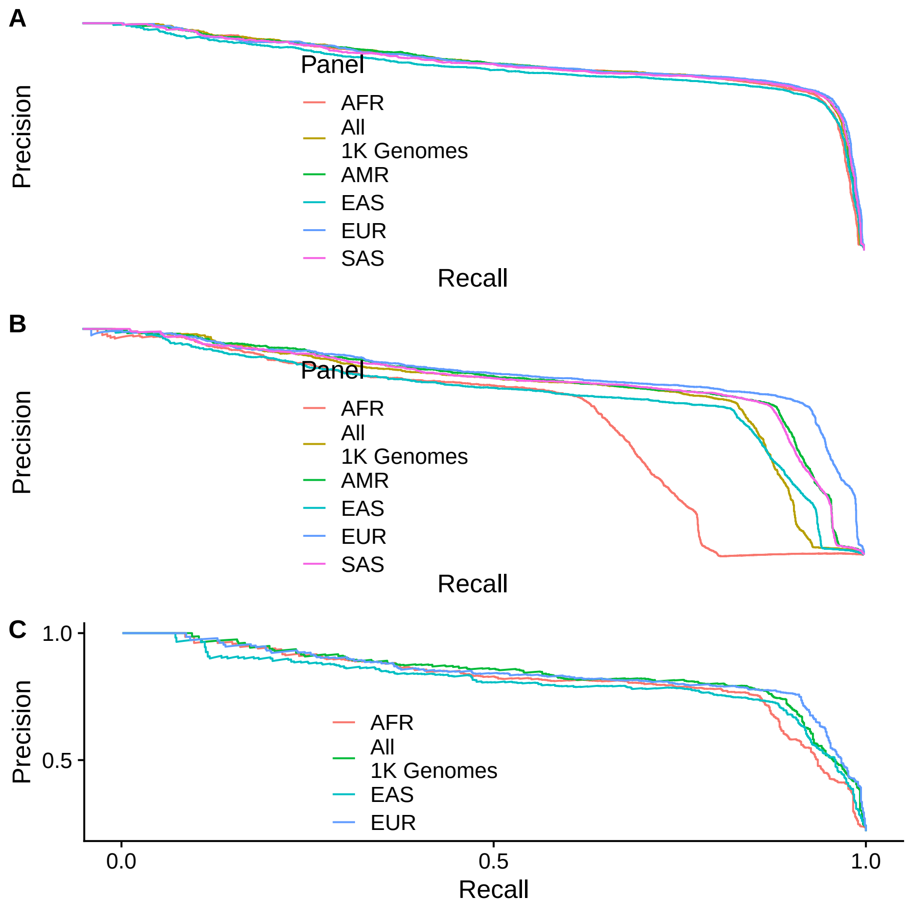

Figure S9: Simulation performance of POEMColoc using different ancestry panels. A: POEMColoc-1 (GWAS imputed). B: POEMColoc-2 (GWAS and eQTL imputed). C: POEMColoc-1 (eQTL imputed). Ancestry panels are taken from unrelated Phase 3 1000 Genomes superpopulation individuals (EUR, EAS, SAS, AFR, AMR) or all 1000 Genomes unrelated individuals (All). EUR is expected to have the best performance because the genotypes used in simulation were from British ancestry individuals from UK Biobank.

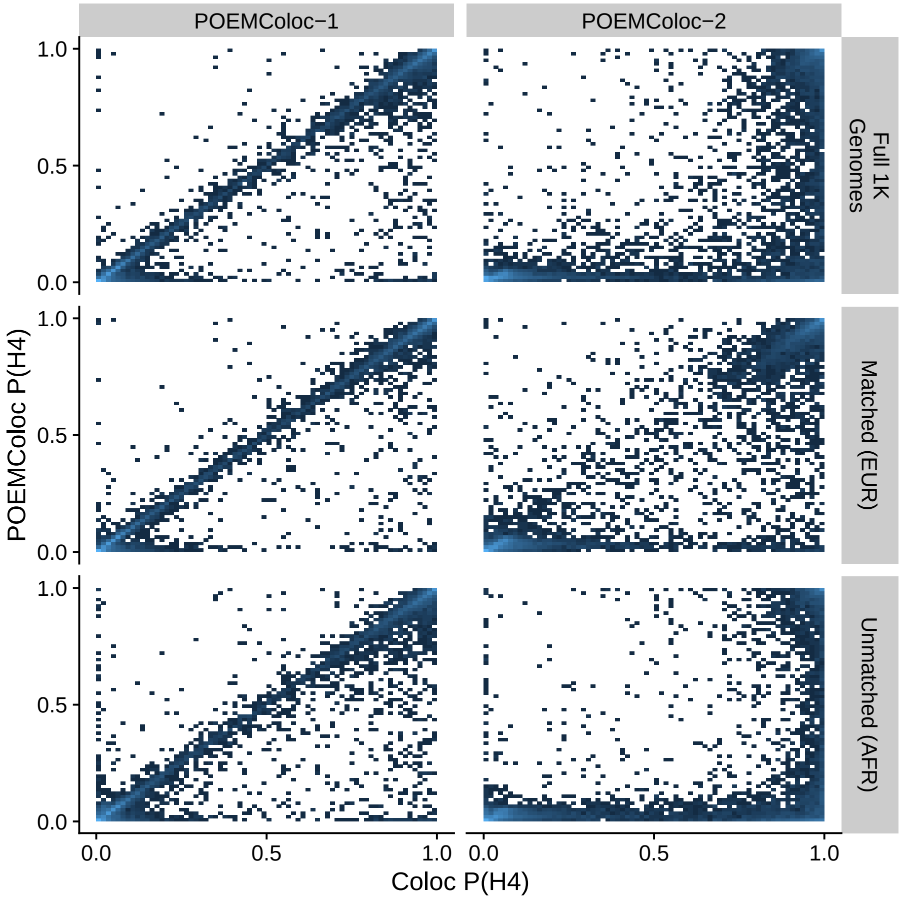

Figure S10: Comparison of estimated colocalization posterior probability $P(H_4)$ between coloc and POEMColoc using ummatched, matched, and mismatched ancestry panels. Left: POEMColoc-1 (GWAS imputed). Right: POEMColoc-2 (GWAS and eQTL imputed).

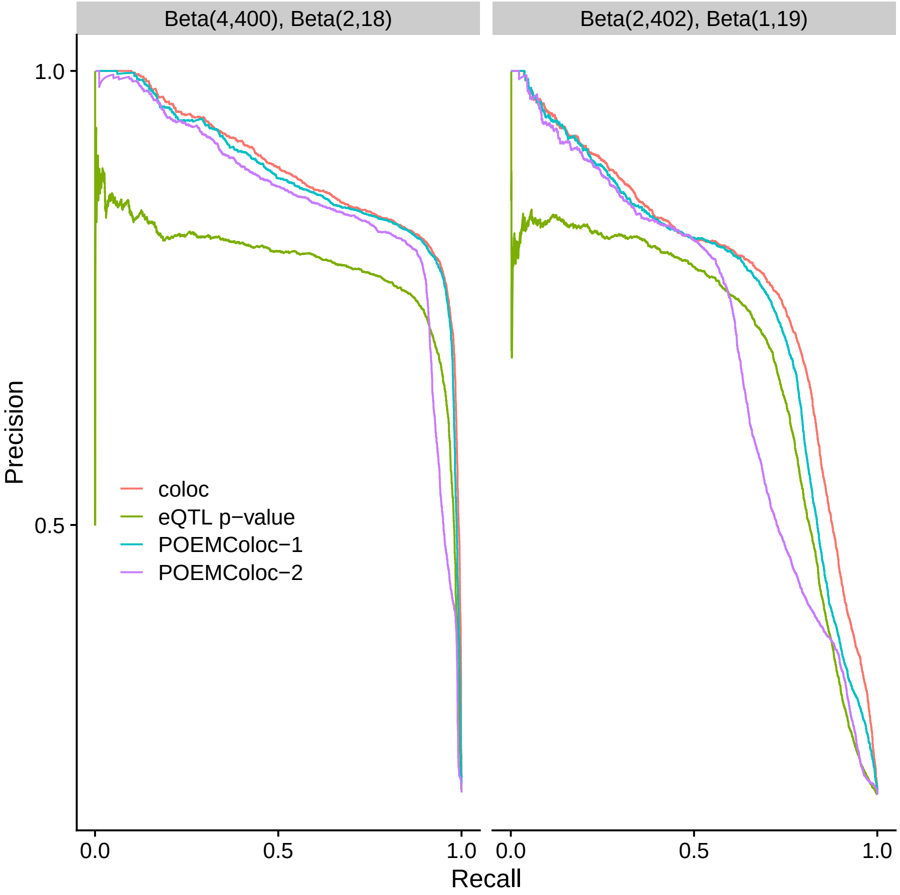

Figure S11: Precision-recall curves for POEMColoc and other methods on datasets simulated using different heritability distributions. Left: Precision recall curve under mean heritability values used in the main text (0.0099 GWAS and 0.1 eQTL). Precision-recall curves are not identical to those from the main text because simulated datasets that could not be analyzed by PICCOLO are also included. Right: Precision-recall curve under halved mean heritability (0.005 GWAS and 0.05 eQTL).

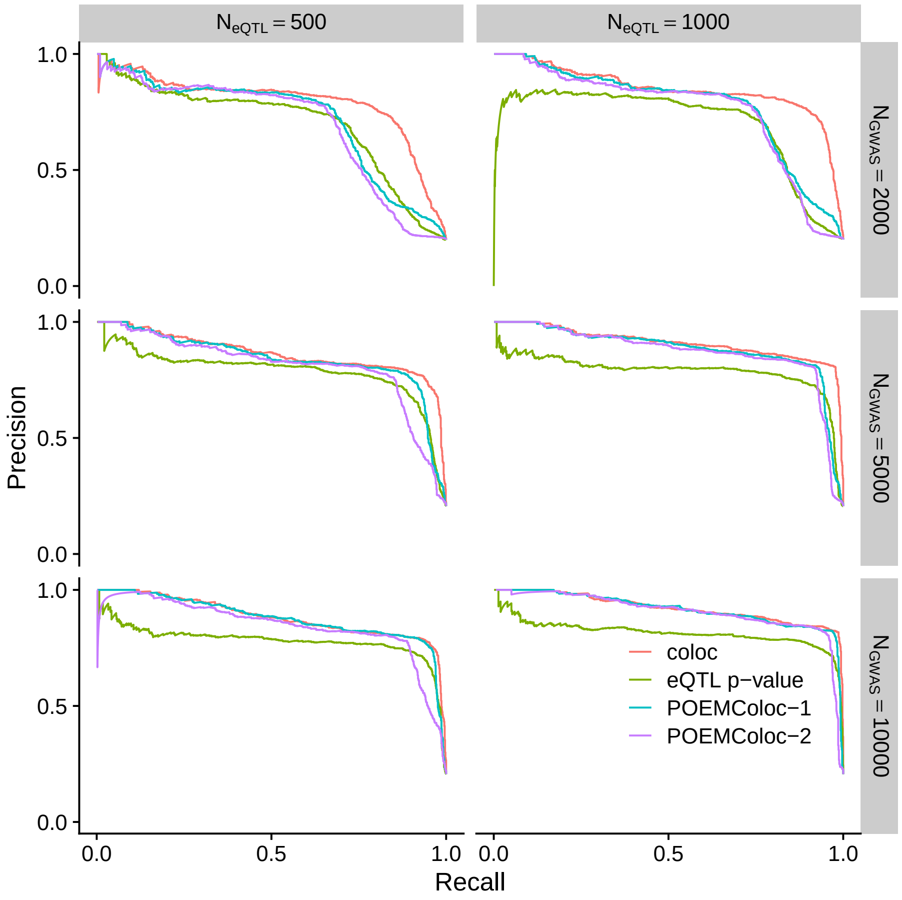

Figure S12: Precision-recall curves for POEMColoc and other methods on simulated GWAS and eQTL datasets by sample size of simulated dataset (mean GWAS heritability 0.0099 and mean eQTL heritability 0.10).

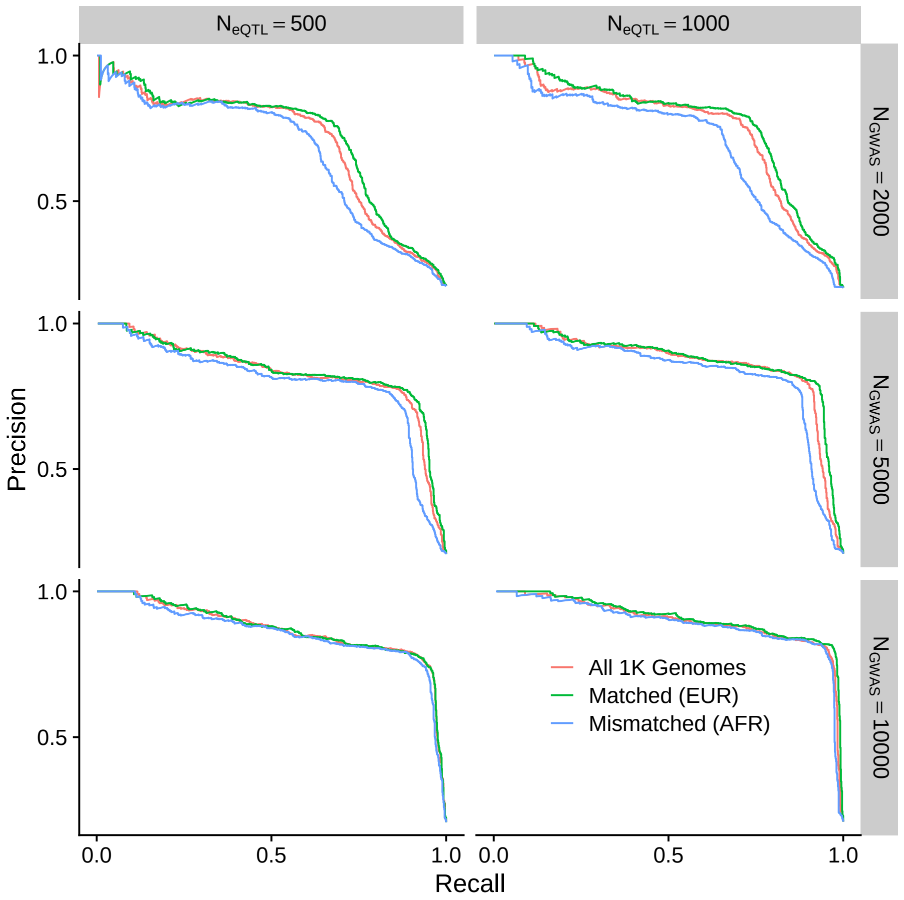

Figure S13: POEMColoc-1 sensitivity to mismatched ancestry panels under different eQTL and GWAS sample size conditions. Precision-recall curves on simulated GWAS and eQTL datasets by sample size of simulated dataset (mean GWAS heritability 0.099 and mean eQTL heritability 0.10).

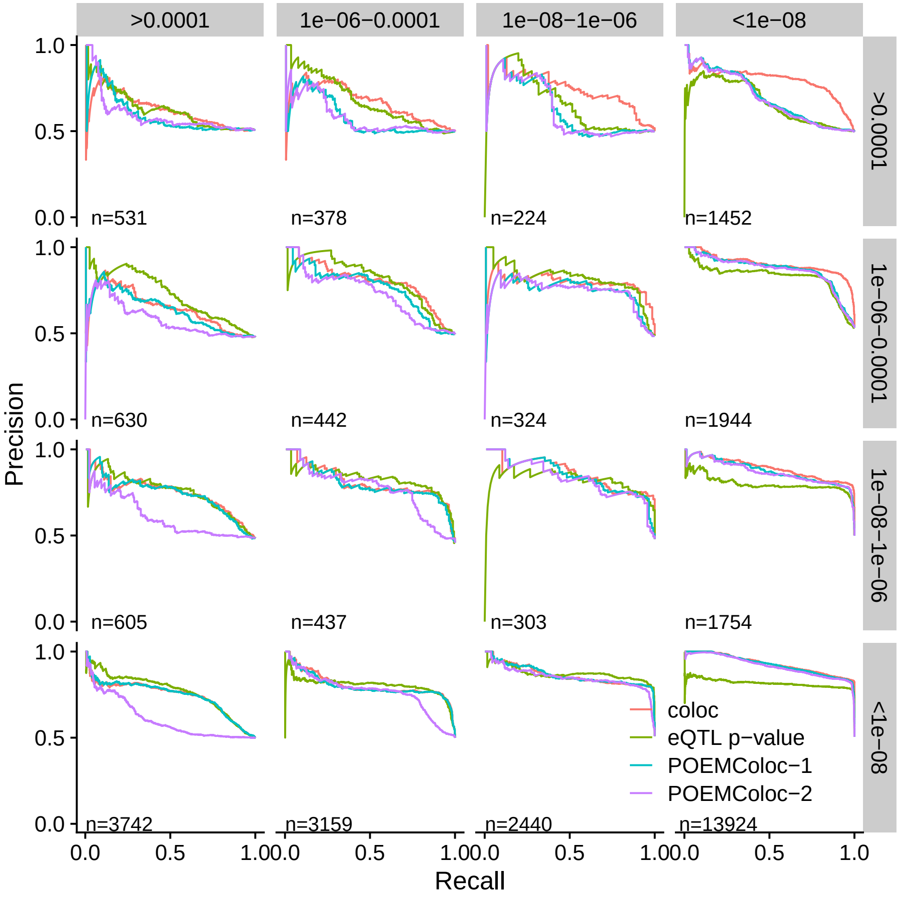

Figure S14: POEMColoc performance relative to other methods in simulations by GWAS (rows) and eQTL (columns) top p-value. Precision-recall curves are computed across 32289 datasets simulated under H_3 and H_4 in the sensitivity analyses to heritability and GWAS and eQTL sample size. Different numbers of simulations are available per bin, number of simulations indicated in figure.

Table S3: Proportion p of SNP-trait pairs with true positive and false negative colocalization that have multiple associations detected by COJO. n is the number analyzed, excluding those where the top SNP was not recognized in the reference panel.

class	n	p
FN	212	0.52
TN	349	0.42
TP	378	0.39

Table S4: Proportion of coloc true positives that are false negatives using POEMColoc-1 in simulation. A coloc true positive is defined as a dataset simulated under colocalization with coloc posterior probability > 0.9. A POEMColoc-1 false negative is a dataset simulated under colocalization with POEMColoc-1 posterior probability less than 0.1. These are defined in order to be comparable to the UK Biobank analysis.

Panel	POEM_FN
African	0.011
All	0.009
Americas	0.009
East Asian	0.007
European	0.004
South Asian	0.006

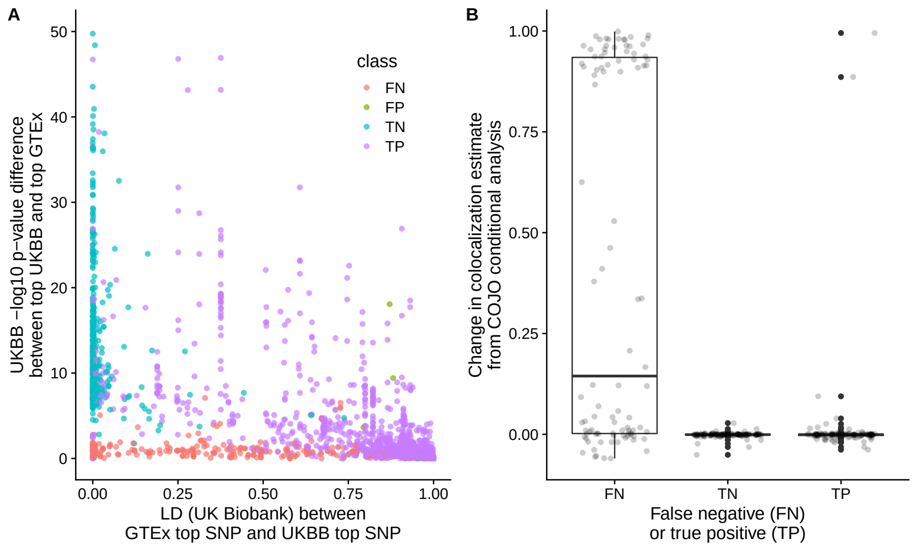

Figure S15: A: LD between GWAS top SNP and GTEx top SNP (x) and difference in GTEx p-value between GTEx top SNP and GWAS top SNP (y) discriminate true positive, false negative, and false positive colocalizations ~~estimated~~ using POEMColoc colocalization of UK Biobank associations and GTEx whole blood expression. Truth is defined to be positive if colocalization probability ~~estimated~~ from the full data is > 0.9 and negative if < 0.1, and positive and negatives from POEMColoc are defined with the same cutoffs. Points in which either method gave a colocalization estimate between 0.1 and 0.9 are excluded. y-axis truncated at 50. B: Reanalyzing datasets using COJO conditional summary statistics for top SNP conditional on all other associations disproportionately effects colocalization probability in false negative datasets compared to true positives.

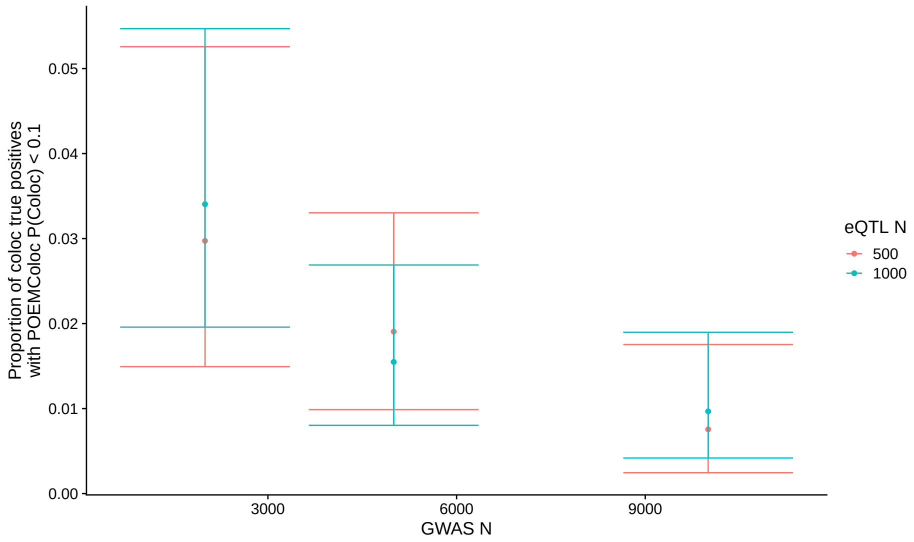

Figure S16: Proportion of POEMColoc-1 false negatives among coloc true positive datasets in simulated datasets under different sample sizes. In simulated datasets, a false negative is defined by $\mathrm{P}(H_4) > 0.9$ using the full data, simulation under hypothesis 4 and $\mathrm{P}(H_4) < 0.1$ using POEMColoc.

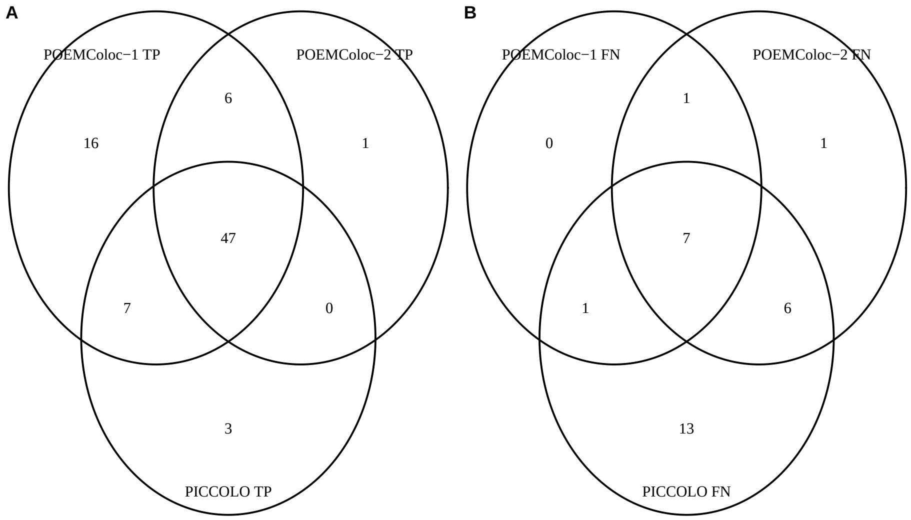

Figure S17: Comparison of POEMColoc-1, POEMColoc-2, and PICCOLO in number of A: true positive colocalizations and B: false negative colocalizations for UK Biobank locus - GTEx eQTL pairs. Truth is defined to be positive if colocalization probability from the full data is > 0.9 and negative if < 0.1, and positive and negatives from POEMColoc and PICCOLO are defined with the same cutoffs. Here, we are only considering the 101 pairs where colocalization was detected using the full data for which all three methods could be run. Note that true positives and false negatives will in general be less than this because frequently one or more methods will yield ambiguous results with colocalization probability between 0.1 and 0.9.

Table S5: Top tissue trait enrichment -log10 p-values detected by POEMColoc-1 and eQTL p-value approach. POEMColoc-1 uses a colocalization cutoff of 0.9. eQTL p-value cutoff of $\sim 10^{-12}$ was chosen such that the total number of gene-trait links between the two methods was the same. Because of the arbitrary choice of thresholds, using the same number of top-ranked links ensures we are using thresholds of similar stringency. All enrichments having p-value 0.05 or lower using either method, and at least 3 genes with significant eQTL or colocalizations for the trait in the tissue are shown.

trait	tissue	POEM	pvalue	Similarity
atrial fibrillation	heart_atrial_appenda	12.00	4.02	0.91
granulocyte percentage of myel	whole_blood	9.85	10.13	0.52
atrial fibrillation	heart_left_ventricle	9.69	3.85	0.62
granulocyte count	whole_blood	8.10	5.50	0.52
neutrophil count;eosinophil co	whole_blood	7.71	5.99	0.48
monocyte percentage of leukocy	whole_blood	7.19	8.25	0.45
neutrophil count;basophil coun	whole_blood	6.24	5.55	0.48
pulse pressure measurement	artery_aorta	5.67	3.31	0.65
neutrophil count	whole_blood	5.54	5.90	0.48
QT interval	heart_left_ventricle	5.50	1.01	0.73
C-C motif chemokine 5 measurem	cells_ebv-transforme	5.38	0.00	NA
migraine without aura	artery_tibial	5.36	4.00	0.00
hypothyroidism	thyroid	5.33	1.60	0.81
ejection fraction measurement	heart_left_ventricle	5.11	3.14	0.74
left ventricular systolic func	heart_left_ventricle	5.11	3.14	0.78
fractional shortening	heart_left_ventricle	5.11	3.14	0.79
migraine disorder	artery_aorta	4.59	1.72	0.23
glucose measurement	thyroid	4.55	2.41	0.40
coronary artery disease	artery_aorta	4.45	3.61	0.54
monocyte count	whole_blood	4.38	3.74	0.45
lipid measurement	liver	4.10	1.09	0.58
Behcet's syndrome	whole_blood	3.98	1.89	0.27
age at menarche;hip bone miner	heart_atrial_appenda	3.87	1.35	0.00
Agouti-related protein measure	thyroid	3.86	3.11	NA
mean platelet volume	whole_blood	3.67	0.89	0.00
systemic scleroderma;systemic	cells_transformed_fi	3.62	3.88	0.65
apolipoprotein A 1 measurement	liver	3.60	4.88	0.62
granulins measurement	liver	3.60	4.88	NA
adolescent idiopathic scoliosi	testis	3.60	0.15	0.20
total cholesterol measurement	liver	3.52	1.10	0.61
repulsive guidance molecule A	liver	3.51	4.88	0.35
mammographic density measureme	adipose_subcutaneous	3.38	1.35	0.00

Table S5: Top tissue trait enrichment -log10 p-values detected by POEMColoc-1 and eQTL p-value approach. POEMColoc-1 uses a colocalization cutoff of 0.9. eQTL p-value cutoff of $\sim 10^{-12}$ was chosen such that the total number of gene-trait links between the two methods was the same. Because of the arbitrary choice of thresholds, using the same number of top-ranked links ensures we are using thresholds of similar stringency. All enrichments having p-value 0.05 or lower using either method, and at least 3 genes with significant eQTL or colocalizations for the trait in the tissue are shown. *(continued)*

trait	tissue	POEM	pvalue	Similarity
femoral neck bone mineral dens	artery_tibial	3.37	1.26	0.27
blood metabolite measurement	stomach	3.31	0.33	NA
myeloid white cell count	whole_blood	3.28	5.55	0.20
migraine without aura	artery_aorta	3.28	0.00	0.32
granulins measurement	minor_salivary_gland	3.23	0.00	NA
rheumatoid arthritis	spleen	3.22	1.90	0.33
systemic scleroderma;systemic	spleen	3.20	0.95	0.51
alcohol drinking	testis	3.20	0.00	0.22
Takayasu arteritis	whole_blood	3.19	0.42	0.18
migraine disorder	artery_tibial	3.18	3.04	0.35
body mass index;hematocrit;glu	liver	3.05	2.13	NA
serum alanine aminotransferase	liver	2.94	0.94	NA
Abdominal Aortic Aneurysm	liver	2.78	4.88	0.15
alcoholic pancreatitis	pancreas	2.58	2.93	0.82
breast size	adipose_subcutaneous	2.50	3.46	0.54
lipoprotein-associated phospho	liver	2.38	1.37	NA
complement factor H measuremen	liver	2.29	3.17	NA
catalase measurement	liver	2.15	3.17	NA
interleukin 18 receptor 1 meas	whole_blood	1.99	3.63	NA
plasminogen activator inhibito	liver	1.97	4.34	NA
C-reactive protein measurement	liver	1.75	3.05	0.50
coronary artery disease	artery_tibial	1.62	3.04	0.49
diastolic blood pressure;smoki	cells_transformed_fi	1.50	5.24	NA
serum gamma-glutamyl transfera	liver	1.43	4.50	NA
prostate specific antigen meas	adrenal_gland	0.85	3.48	NA
systolic blood pressure;smokin	cells_transformed_fi	0.70	4.34	NA
coffee consumption;cups of cof	thyroid	0.48	5.07	0.19
progranulin measurement	liver	0.34	3.82	NA
vital capacity	brain_putamen_basal_	0.28	3.02	0.00
systolic blood pressure;alcoho	cells_transformed_fi	0.26	3.35	NA
QRS complex;QRS amplitude	thyroid	0.16	8.28	0.22
bipolar disorder;schizophrenia	esophagus_mucosa	0.00	4.16	0.00

Table S5: Top tissue trait enrichment -log10 p-values detected by POEMColoc-1 and eQTL p-value approach. POEMColoc-1 uses a colocalization cutoff of 0.9. eQTL p-value cutoff of $\sim 10^{-12}$ was chosen such that the total number of gene-trait links between the two methods was the same. Because of the arbitrary choice of thresholds, using the same number of top-ranked links ensures we are using thresholds of similar stringency. All enrichments having p-value 0.05 or lower using either method, and at least 3 genes with significant eQTL or colocalizations for the trait in the tissue are shown. *(continued)*

trait	tissue	POEM	pvalue	Similarity
response to bronchodilator;for	muscle_skeletal	0.00	3.55	NA
response to bronchodilator;FEV	muscle_skeletal	0.00	3.55	NA
smoking status measurement;uri	colon_transverse	0.00	3.45	NA
intracranial volume measuremen	brain_amygdala	0.00	3.12	0.25
coronary heart disease;lipopro	liver	NA	4.88	NA
coronary heart disease;acute c	liver	NA	4.88	NA
t-tau measurement	thyroid	NA	3.10	NA

Table S6: Approved drug mechanisms with colocalization.

indication	symbol	Trait	sim
Psoriasis	ITGAL	Crohn's disease;psoriasis;ulce	1.00
Psoriasis	PDE4A	Crohn's disease;psoriasis;ulce	1.00
Crohn Disease	PTGS2	Crohn's disease	1.00
Psoriasis	IL23A	psoriasis	1.00
Schizophrenia	DRD2	schizophrenia	1.00
Depression	DRD2	depressive symptom measurement	1.00
Arthritis, Rheumatoi	IL6R	rheumatoid arthritis	1.00
Psoriasis	IL6R	Crohn's disease;psoriasis;ulce	1.00
Arthritis, Juvenile	IL6R	systemic juvenile idiopathic a	1.00
Asthma	ADORA1	asthma	1.00
Atrial Fibrillation	SCN5A	atrial fibrillation	1.00
Colitis, Ulcerative	PTGS2	Crohn's disease	0.90
Factor X Deficiency	F10	prothrombin time measurement	0.88
Arthritis, Psoriatic	PDE4A	Crohn's disease;psoriasis;ulce	0.87
Infertility, Female	LHCGR	polycystic ovary syndrome	0.84
Hyperlipidemias	LPL	triglyceride measurement	0.83
Inflammatory Bowel D	PTGS2	Crohn's disease	0.83
Hypercholesterolemia	NPC1L1	total cholesterol measurement	0.82
Hypercholesterolemia	HMGCR	total cholesterol measurement	0.82
Hypercholesterolemia	PCSK9	total cholesterol measurement	0.82
Hypercholesterolemia	PPARA	total cholesterol measurement	0.82

Table S6: Approved drug mechanisms with colocalization. *(continued)*

indication	symbol	Trait	sim
Hypercholesterolemia	LPL	triglyceride measurement;high	0.82
Psychotic Disorders	DRD2	schizophrenia	0.82
Hyperlipoproteinemia	LPL	triglyceride measurement	0.81
Tachycardia, Ventric	SCN5A	PR interval	0.81
Disseminated Intrava	F10	prothrombin time measurement	0.81
Disseminated Intrava	F2	prothrombin time measurement	0.81
Arthritis, Juvenile	IL1R1	Crohn's disease;psoriasis;ulce	0.80
Thyroid Neoplasms	FGFR3	bladder carcinoma	0.80
Arrhythmias, Cardiac	KCNH2	QT interval	0.80
Arrhythmias, Cardiac	SCN5A	PR interval	0.80
Hypophosphatasia	ALPL	vitamin B6 measurement	0.80
Arthritis	PDE4A	Crohn's disease;psoriasis;ulce	0.80
Bipolar Disorder	DRD2	schizophrenia	0.79
Arthritis, Rheumatoi	IL1R1	Crohn's disease;psoriasis;ulce	0.79
Hemophilia B	F7	prothrombin time measurement	0.79
Hemophilia B	F10	prothrombin time measurement	0.79
Hemophilia B	F2	prothrombin time measurement	0.79
Hyperlipidemias	NPC1L1	total cholesterol measurement	0.79
Hyperlipidemias	HMGCR	total cholesterol measurement	0.79
Hyperlipidemias	PCSK9	total cholesterol measurement	0.79
Hyperlipidemias	PPARA	total cholesterol measurement	0.79
Hyperlipoproteinemia	NPC1L1	total cholesterol measurement	0.79
Hyperlipoproteinemia	APOB	total cholesterol measurement	0.79
Hyperlipoproteinemia	HMGCR	total cholesterol measurement	0.79
Hyperlipoproteinemia	PCSK9	total cholesterol measurement	0.79
Ventricular Fibrilla	KCNH2	QT interval	0.79
Ventricular Fibrilla	SCN5A	PR interval	0.79
Multiple Sclerosis,	IL2RA	multiple sclerosis	0.78
Depressive Disorder,	DRD2	neuroticism measurement	0.78
Dermatitis, Atopic	PDE4A	Crohn's disease;psoriasis;ulce	0.78
Hypertension	ADRB1	systolic blood pressure	0.77
Hypertension	AGT	systolic blood pressure	0.77
Hypertension	ACE	diastolic blood pressure	0.77
Hypertension	NOS3	diastolic blood pressure	0.77
Thyroid Neoplasms	TSHR	Graves disease	0.76
Hypotension	AGT	mean arterial pressure	0.76
Bronchitis, Chronic	ADORA1	asthma	0.75
Atherosclerosis	LPL	triglyceride measurement;high	0.75
Neutropenia	CSF3	neutrophil count	0.75
Depressive Disorder,	MTNR1B	conduct disorder	0.75

Table S6: Approved drug mechanisms with colocalization. *(continued)*

indication	symbol	Trait	sim
Chest Pain	SCN5A	PR interval	0.74
Hypersensitivity	ADORA1	asthma	0.74
Constriction, Pathol	PDE4A	Crohn's disease;psoriasis;ulce	0.74
Endocrine System Dis	TPO	hypothyroidism	0.74
Atrial Fibrillation	KCNH2	QT interval	0.74
Hypertriglyceridemia	HMGCR	total cholesterol measurement	0.73
Hypertriglyceridemia	PPARA	total cholesterol measurement	0.73
Myelodysplastic Synd	DNMT1	myeloid white cell count	0.73
Dermatitis, Atopic	IL4R	eosinophil count	0.73
Hemophilia A	F7	prothrombin time measurement	0.73
Hemophilia A	F10	prothrombin time measurement	0.73
Hemophilia A	F2	prothrombin time measurement	0.73
Hypogonadism	LHCGR	polycystic ovary syndrome	0.73
Leukemia, Myeloid, A	FLT3	granulocyte percentage of myel	0.73
Leukemia, Myeloid, A	DNMT1	granulocyte count	0.73
Hemorrhage	F7	prothrombin time measurement	0.73
Hemorrhage	F10	prothrombin time measurement	0.73
Hemorrhage	F2	prothrombin time measurement	0.73
Pulmonary Disease, C	ADORA1	asthma	0.73
Atherosclerosis	HMGCR	total cholesterol measurement	0.71
Atherosclerosis	PPARA	total cholesterol measurement	0.71
Breast Neoplasms	ERBB2	age at menopause	0.71
Diabetes Mellitus, T	LPL	triglyceride measurement;high	0.71
Kidney Neoplasms	FGFR3	bladder carcinoma	0.70
Leukemia, Myelogenou	DNMT1	myeloid white cell count	0.70

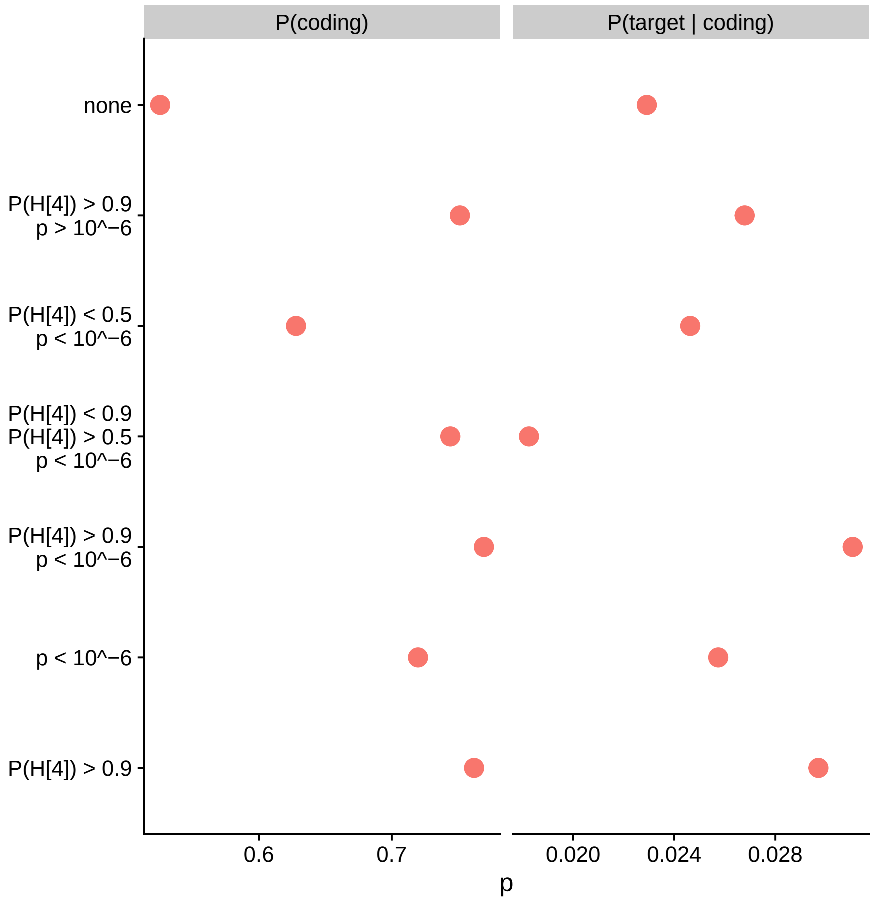

Figure S18: Proportion of gene-trait pairs eQTL p-value of the GWAS top SNP and colocalization posterior probability $\mathrm{P}(H_4)$ meeting cutoffs that involve coding genes (A) and approved drug targets conditional on being coding (B). xMHC genes were excluded from both the numerator and the denominator of B because they are unavailable in approved drug target dataset.

References

[1] J. Yang, S. H. Lee, M. E. Goddard, and P. M. Visscher, "Gcta: a tool for genome-wide complex trait analysis," *The American Journal of Human Genetics*, vol. 88, no. 1, pp. 76–82, 2011.

[2] T. Saito and M. Rehmsmeier, "Precrec: fast and accurate precision–recall and roc curve calculations in r," *Bioinformatics*, vol. 33, no. 1, pp. 145–147, 2017.

[3] A. Canty and B. D. Ripley, *boot: Bootstrap R (S-Plus) Functions*, 2019. R package version 1.3-24.

[4] K. Boyd, K. H. Eng, and C. D. Page, "Area under the precision-recall curve: point estimates and confidence intervals," in *Joint European conference on machine learning and knowledge discovery in databases*, pp. 451–466, Springer, 2013.

[5] C. Giambartolomei, D. Vukcevic, E. E. Schadt, L. Franke, A. D. Hingorani, C. Wallace, and V. Plagnol, "Bayesian test for colocalisation between pairs of genetic association studies using summary statistics," *PLoS genetics*, vol. 10, no. 5, p. e1004383, 2014.

[6] C. Giambartolomei, J. Zhenli Liu, W. Zhang, M. Hauberg, H. Shi, J. Boocock, J. Pickrell, A. E. Jaffe, C. Consortium, B. Pasaniuc, *et al.*, "A bayesian framework for multiple trait colocalization from summary association statistics," *Bioinformatics*, vol. 34, no. 15, pp. 2538–2545, 2018.
